# Effect of Regulatory Architecture on Broad versus Narrow Sense Heritability

**DOI:** 10.1371/journal.pcbi.1003053

**Published:** 2013-05-09

**Authors:** Yunpeng Wang, Jon Olav Vik, Stig W. Omholt, Arne B. Gjuvsland

**Affiliations:** 1 Centre for Integrative Genetics (CIGENE), Department of Animal and Aquacultural Sciences, Norwegian University of Life Sciences, Ås, Norway; 2 Centre for Integrative Genetics (CIGENE), Department of Mathematical Sciences and Technology, Norwegian University of Life Sciences, Ås, Norway; 3 NTNU Norwegian University of Science and Technology, Department of Biology, Centre for Biodiversity Dynamics, Realfagsbygget, NO-7491 Trondheim, Norway; University of Washington, United States of America

## Abstract

Additive genetic variance (*V_A_*) and total genetic variance (*V_G_*) are core concepts in biomedical, evolutionary and production-biology genetics. What determines the large variation in reported *V_A_*/*V_G_* ratios from line-cross experiments is not well understood. Here we report how the *V_A_*/*V_G_* ratio, and thus the ratio between narrow and broad sense heritability (*h^2^*/*H^2^*), varies as a function of the regulatory architecture underlying genotype-to-phenotype (GP) maps. We studied five dynamic models (of the cAMP pathway, the glycolysis, the circadian rhythms, the cell cycle, and heart cell dynamics). We assumed genetic variation to be reflected in model parameters and extracted phenotypes summarizing the system dynamics. Even when imposing purely linear genotype to parameter maps and no environmental variation, we observed quite low *V_A_*/*V_G_* ratios. In particular, systems with positive feedback and cyclic dynamics gave more non-monotone genotype-phenotype maps and much lower *V_A_*/*V_G_* ratios than those without. The results show that some regulatory architectures consistently maintain a transparent genotype-to-phenotype relationship, whereas other architectures generate more subtle patterns. Our approach can be used to elucidate these relationships across a whole range of biological systems in a systematic fashion.

## Author Summary

The *broad-sense heritability* of a trait is the proportion of phenotypic variance attributable to genetic causes, while the *narrow-sense heritability* is the proportion attributable to additive gene effects. A better understanding of what underlies variation in the ratio of the two heritability measures, or the equivalent ratio of additive variance *V_A_* to total genetic variance *V_G_*, is important for production biology, biomedicine and evolution. We find that reported *V_A_*/*V_G_* values from line crosses vary greatly and ask if biological mechanisms underlying such differences can be elucidated by linking computational biology models with genetics. To this end, we made use of models of the cAMP pathway, the glycolysis, circadian rhythms, the cell cycle and cardiocyte dynamics. We assumed additive gene action from genotypes to model parameters and studied the resulting GP maps and *V_A_*/*V_G_* ratios of system-level phenotypes. Our results show that some types of regulatory architectures consistently preserve a transparent genotype-to-phenotype relationship, whereas others generate more subtle patterns. Particularly, systems with positive feedback and cyclic dynamics resulted in more non-monotonicity in the GP map leading to lower *V_A_*/*V_G_* ratios. Our approach can be used to elucidate the *V_A_*/*V_G_* relationship across a whole range of biological systems in a systematic fashion.

## Introduction

The broad-sense heritability of a trait, 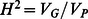, is the proportion of phenotypic variance attributable to genetic causes, while the narrow-sense heritability 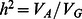, is the proportion attributable to additive gene action. The total genetic variance 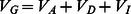 includes the variance explained by intra-locus dominance (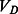) and inter-locus interactions (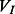). The reasons for and importance of this non-additive genetic variance that distinguishes the two heritability measures has been subject to substantial controversy for more than 80 years (e.g., [1]–[6]). It was recently shown through statistical arguments that for traits with many loci at extreme allele frequencies, much of the genetic variance becomes additive with *h^2^*/*H^2^* (or equivalently *V_A_*/*V_G_*) typically >0.5 [3]. In populations with intermediate allele frequencies, such as controlled line crosses, lower *V_A_*/*V_G_* ratios are often reported [7], [8]. This is illustrated in Table 1, which summarizes estimated *V_A_*/*V_G_* ratios from a collection of studies on such populations. This wide range of *h^2^*/*H^2^* ratios reported for line crosses cannot be explained by an allele-frequency argument, and putative explanations must be based on how the regulatory architecture of the underlying biological systems shape the genotype-phenotype (GP) map.

**Table 1:** Examples of reported *V_A_*/*V_G_* ratios of from line-crossing experiments.

Species	Type of traits	Number of traits	Ref.	Min *V_A_*/*V_G_*	Max *V_A_*/*V_G_*	Mean *V_A_*/*V_G_*
Chicken	Growth, weight	17	[45]	0.03	0.71	0.29
Mouse	Hyperoxic survival	1	[46]	-	-	0.46
*Drosophila melanogaster*	Locomotor behavior	1	[47]	-	-	0.31
*Drosophila melanogaster*	Olfactory behavior	1	[48]	-	-	0.64
Upland cotton	Nematode resistance	1	[49]	-	-	0.79
Melon	Fruit color and maturity	2	[50] a	0.55	0.58	0.57
Maize	Morphological traits	17	[51] b	0.13	1.1	0.59
Maize	Leaf spot resistance	5	[52]	0.51	0.95	0.76
*Arabidopsis thaliana*	Flowering and morphology and	22	[53] c	0.58	1.05	0.76
Eggplant	Callus related traits	4	[54]	0.42	0.93	0.73

a. Average of ratios from generation mean analysis and variance component method.

b. Using average of inbred and hybrid line estimates of h^2^ and H^2^.

c. Using estimated h^2^ and H^2^ from triple test cross.

It is important to understand the causal underpinnings of the observed variation in *h^2^/H^2^* ratios within and between biological systems for several reasons. In human quantitative genetics, where twin studies are commonly used, most heritability estimates refer to *H^2^* [9]. In cases where *h^2^/H^2^* is low, this can lead to unrealistic expectations about how much of the underlying causative variation may be located by linear QTL detection methods [6]. On the other hand, low narrow sense heritability for a given complex trait does not necessarily imply that the environment determines much of the variation. In evolutionary biology, additive variance is the foremost currency for evolutionary adaptation and evolvability. Important questions in this context are for example (i) to which degree is there selection on the regulatory anatomies themselves to maintain high additive variance, (ii) are there organizational constraints in building adaptive systems such that in some cases a low *h^2^*/*H^2^* ratio must of necessity emerge while the proximal solution is still selected for? Moreover, in production biology with genetically modified, sexually reproducing organisms, one would like to ensure that the modifications would be passed over to future generations in a fully predictable way. Thus, one would like to ensure that the modification becomes highly heritable in the narrow sense.

As a step towards a physiologically grounded understanding of the variation of the *h^2^*/*H^2^* relationship across biological systems or processes, we posed the question: Are there regulatory structures, or certain classes of phenotypes, more likely to generate low *V_A_*/*V_G_* ratios than others? Addressing this question requires the linking of genetic variation to computational biology in a population context (e.g., [10]–[19]), so-called causally-cohesive genotype-phenotype (cGP) modeling [15], [17], [18]. We applied this approach to five well-validated computational biology models describing, respectively, the glycolysis metabolic pathway in budding yeast [20], the cyclic adenosine monophosphate (cAMP) signaling pathway in budding yeast [21], the cell cycle regulation of budding yeast [22], the gene network underlying mammalian circadian rhythms [23], and the ion channels determining the action potential in mouse heart myocytes [24] These models differ in their regulatory architecture; below, we show that they also differ in the range of *V_A_*/*V_G_* ratios that they can exhibit. In particular, positive feedback regulation and oscillatory behavior seem to dispose for low *V_A_*/*V_G_* ratios. The results suggest that our approach can be used in a generic manner to probe how the *h^2^*/*H^2^* ratio varies as a function of regulatory anatomy.

## Methods

### Simulations of cGP models

The five cGP models were built and analyzed with the *cgptoolbox* (http://github.com/jonovik/cgptoolbox) an open-source Python package developed by the authors; further source code specific to the simulations in this paper is available on request. In the following we describe the three main parts of the workflow: (i) the mapping from genotypes to parameters, (ii) the mapping from parameters to phenotypes, i.e. solving the dynamic models and (iii) the setup of Monte-Carlo simulations combining the two mappings (Figure S1). For each model, we briefly describe its origins, the software used to solve it, which parameters were subject to genetic variation, what phenotypes were recorded, and criteria for omitting outlying datasets. Figures S2, S3, S4, S5, S6 shows graphical representations of the five model systems and Text S1 contains more detailed descriptions of all five models.

#### Genotype to parameter mapping

For each model, the following procedure was repeated 1000 times (see also “Monte Carlo simulations” below) for different selections of parameters to be subjected to simulated genetic variation. We started by sampling three polymorphic loci, each determining one or two parameters in the dynamic model. Tables of eligible loci with corresponding parameters and their baseline values are listed in Table S1, S2, S3, S4, S5, corresponding to the cAMP, glycolysis, cell cycle, circadian and action potential models respectively. Heritable variation in a chosen parameter was generated for a single biallelic locus with allele indexes 0 and 1 in the following manner. First, two numbers *r_1_* and *r_2_* were sampled uniformly in the interval [0.7, 1.3]. The parameter value for a homozygote 00 was set to 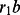 where *b* is the baseline value, for a homozygote 11 the parameter value was 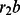. The heterozygous genotype 01 was assigned the average of the two homozygotes 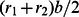, resulting in an additive mapping from genotypes to parameter values.

#### cAMP model

**Figure 1:**
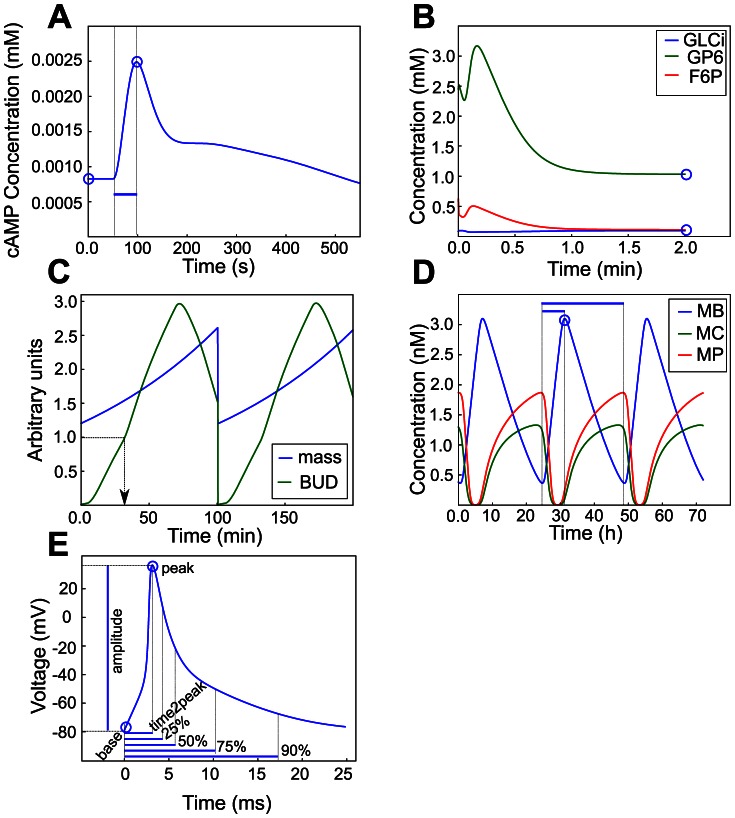
Phenotypes derived from the cGP models. Graphical illustration of the phenotypes recorded for the five cGP models studied. Time courses (state variable on y-axis, time on x-axis) for the baseline parameter set are displayed for all five models. **A.** In the absence of external glucose all state variables (concentration of cAMP is shown) in the cAMP model [21] converge to a stable steady state (blue circle on y-axis). After addition of external glucose (5 mM added at time 50) we see a rapid change followed by a slow return to the original steady state. In addition to the original steady state, the extremal concentration (top blue circle) as well as the time to reach the extremum (blue line) was recorded as phenotypes. **B.** Metabolite concentrations (internal glucose (GLCi), glucose-6-phospate (G6P) and fructose-6-phospate (F6P) are shown) in the glycolysis model [20] all converge to a stable steady state, indicated by open circles. The steady state concentrations for 13 metabolites were recorded as phenotypes from this model. **C.** For the cell cycle model [22] we recorded the peak level and the time from bottom to peak as for the circadian model (Figure 1D), and in addition we recorded cell cycle events such as bud emergence at the time when [BUD] = 1 indicated by the black arrow. **D.** mRNA and protein concentrations (mRNA for *Bmal1* (MB), *Cry* (MC) and *Per* (MP) are shown) in the circadian model [23] converge to a limit cycle. In addition to the period of oscillation (long blue line) for each of the 16 variables the peak level (open blue circle) as well as the time from bottom to peak (short blue line) were recorded as phenotypes. **E.** We used the base level, peak level, amplitude, time to peak, and time to 25%, 50%, 75% and 90% recovery of the action potential and calcium transient as cell level phenotypes of the action potential model [24]. An action potential is shown in the figure.

The model of the complete cAMP signaling pathway in *S. cerevisiae* [21] taking the external glucose level as input was downloaded as SBML code (http://www.biomedcentral.com/content/supplementary/1752-0509-3-70-s1.xml) and integrated using PySCeS [25]. Genetic variation was introduced on association/dissociation and phosphorylation/dephosphorylation rates of signal proteins (see Figure S2 and Table S1). The initial steady state concentrations before adding external glucose, the peak values after adding glucose and the time taken to reach peak values of cellular proteins were recorded as phenotypes (see Figure 1A for phenotype illustration and Table S6 for phenotype descriptions).

#### Glycolysis model

The model published by Teusink et al. [20] describes glycolysis in *S. cerevisiae* through the kinetics of 13 glycolytic enzymes determining the fluxes of metabolite state variables. Genetic variation was introduced on maximal reaction rates for the enzymes (see Figure S3 and Table S2). We downloaded the model from the BioModels database (http://www.ebi.ac.uk/biomodels-main/BIOMD0000000064) in SBML L2 V1, and solved it with PySCeS [25] to find the stable steady state concentrations of metabolites, which were used as phenotypes (see Figure 1B and Table S7). Datasets were discarded if one or more of the genotypes did not give a stable steady state, as can happen due to a saddle-node bifurcation [26].

#### Cell cycle model

The model of the consensus control mechanisms of the cell cycle in *S. cerevisae* modeled by algebraic/differential equations that describe the continuous changes in state variables and discontinuities due to cellular events [22] was obtained from the CellML repository (http://models.cellml.org/workspace/chen_calzone_csikasznagy_cross_novak_tyson_2004). Genetic variation was introduced on the production and decay rates of various proteins (see Figure S4 and Table S3). The published model contains reset rules (events) for both parameters and state variables, but the CellML implementation only includes the parameter (*k*_mad2_, *k*_bub2_ and *k*_lte1_) rules. Reset rules for state variables [BUD], [SPN], and [ORI] as described in the model paper, were implemented by solving the model with rootfinding for the relevant variables. The model was numerically integrated using the CVODE solver [27] until convergence of cell division time, cell cycle events. The peak levels and time to peak levels for the cytosolic protein concentrations, together with the timing of cell division events were recorded as phenotypes (see Figure 1C for phenotype illustrations and Table S8 for phenotype descriptions).

#### Circadian model

The model of the mammalian circadian clock published by Leloup and Goldbeter [23] describes the dynamics of mRNA and proteins of three genes in the cytosol and nucleus. Genetic variation was introduced on mRNA decay rates (see Figure S5 and Table S4). The model was downloaded from CellML repository (http://models.cellml.org/workspace/leloup_goldbeter_2004) and integrated using CVODE [27] until convergence to its limit cycle. As phenotypes we used the bottom levels and time to from bottom level to peak value of the concentrations of mRNAs, proteins and protein complexes. In addition, we recorded the period of oscillations (see Figure 1D for phenotype illustrations and Table S9 for phenotype descriptions).

#### Action potential model

The model of [24] is an extension of [28] and describes the action potential and calcium transient of a mouse heart muscle cell. We obtained CellML code from the authors and the file is available as supplementary material in [17]. Numerical integration was done using CVODE [27]. Genetic variation was introduced on the maximal conductances of ion channels and pump affinities (see Figure S6 and Table S5). Phenotypes were generated by simulated regular pacing as done in [17], [18], with a stimulus potassium current of −15 V/s was lasting for 3 ms at the start of each stimulus interval. The model was simulated to convergence as described in [17]; datasets that failed to converge were discarded. The initial level, peak level, amplitude, and time to 25, 50, 75 and 90% recovery of the action potential and calcium transient were recorded as the cell level phenotypes (see Figure 1E for phenotype illustrations and Table S10 for phenotype descriptions).

#### Monte Carlo simulations

For each model we performed 1000 Monte Carlo simulations as follows (see Figure S1 for an illustration). We first sampled three polymorphic loci for introduction of genetic variation and sampled the genotype-to-parameter map as described above. Then all 27 possible three-locus genotypes were enumerated, mapped into 27 parameter sets and for each parameter set the dynamic model was solved and phenotypes (as described above and in Figure 1) were obtained. To avoid artifacts arising from numerical noise datasets with low variability were omitted from the genetic analysis. Absolute variability was measured as the difference between the maximum and minimum values of a phenotype in a dataset, and relative variability as the ratio of the absolute variation to the mean phenotype of the same dataset. The threshold values for each phenotype and the number of datasets exceeding the thresholds are listed in Tables S6, S7, S8, S9, S10, for the cAMP, glycolysis, cell cycle, circadian and action potential models, respectively.

### Statistical analysis

#### Decomposition of genetic variance

A single Monte Carlo simulation results in genotype-to-phenotype maps comprised by 27 genotypic values (i.e. the phenotype values corresponding to the 27 genotypes) for a given phenotype. We used the NOIA framework [29] to compute the resulting genetic variance (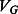) in a hypothetical F_2_ population and decompose it into additive (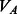) and non-additive components (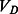 and 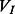). This was done with the function *linearGPmapanalysis* in the R package *noia* (http://cran.r-project.org/web/packages/noia/) version 0.94.1.

#### Monotonicity of GP-maps

We build on the definitions of monotonicity and the indexing of alleles introduced in [30]. Given a simulated GP map with 27 genotypic values we measured the degree of order-breaking for a particular locus *k* by the allele substitution effects at that locus. For a fixed background genotype at all other loci (as indicated in eq. (14) in [30]), we computed the difference in genotypic value when substituting a 0-allele with a 1-allele (i.e. when going from 00 to 01 or from 01 to 11 at locus *k*). We collected substitution effects across all 9 background genotypes to compute *N*, the sum of all negative substitution effects, and *A*, the sum of absolute values of all substitution effects. If the GP map is monotone for locus *k* then 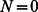, and if it is order-breaking for locus *k* the 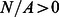.

## Results/Discussion

### System classification and phenotype dimensionality

The five cGP models studied in this work differ in several ways, both in their function and the underlying network structure. The glycolysis and cAMP models are both pathway models with an acyclic series of reactions transforming inputs to outputs. The glycolysis model [20] is more advanced than the metabolic models in earlier genetically oriented studies (e.g., [3], [31], [32]) as it contains many different types of enzyme kinetics as well as negative feedback regulation of some enzyme activities through product inhibition. The cAMP model [21] contains a number of negative feedback loops, but overall it also has a clear pathway structure where the glucose signal is relayed from G-proteins to cAMP to the target kinase PKA. Both these two models have in common relatively simple dynamics with solutions converging to a stable steady state (Figure 1A and B).

In contrast, the three other models show cyclic behavior resulting from an interplay between positive and negative feedback loops (Figure 1 C–E). However, there are clear differences between these models too. The heart cell model [24] is an excitable system with feedback mechanisms including calcium-induced calcium release and several voltage-dependent ion channels. In contrast to pacemaker cells, it relies on external pacing to initiate the action potential. The circadian rhythm model [23], [33] is a gene expression network with intertwined positive and negative transcriptional feedback loops, giving a limit cycle oscillator with sustained oscillations even in continuous darkness. The cell cycle model [22] centers around a positive feedback loop between B-type cyclins in association with cyclin dependent kinase and inhibitors of the cyclin dependent kinase, which establishes a hysteresis loop causing the cell cycle to emerge from transitions between the two alternative stable steady states.

**Figure 2:**
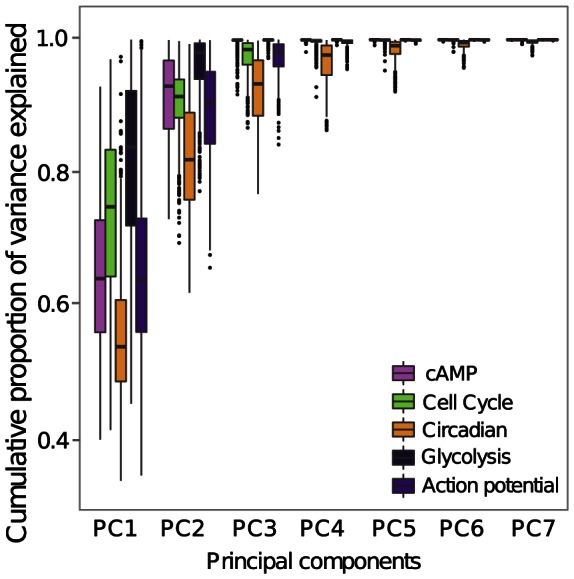
Dimensionality of phenotypic variation. The proportion of total phenotypic variation explained (y axis) versus the number of principal components (x axis) across all five cGP models (colour coded). For each Monte Carlo data set the 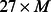 matrices containing the full genotype-phenotype map for all *M* recorded phenotypes was standardized to unit variance before principal components analysis. Each boxplot summarizes explained variance for close to 1000 Monte Carlo simulations.

This crude classification of the five cGP models into pathway models and more complex regulatory systems is clearly reflected in the effective dimensionality of the phenotypes arising in our Monte Carlo simulations. We studied the phenotypic dimensionality for all five cGP models by Principal Component Analysis (PCA) for each Monte Carlo simulation (Figure 2). Across all simulated datasets, 95% of phenotypic variation of the glycolysis and cAMP models can be explained by the first 3 principal components, the cell cycle and heart cell models require the first 5 principal components, and 7 components are required for the circadian model. Since the genotype-to-parameter maps are additive for all five models, these differences in the effective dimensionality of high-level phenotypes indicate that the mappings from parameters to phenotypes are simpler for the pathway models than the other three models. This, together with results on the effect of positive feedback on statistical epistasis in gene regulatory networks [11], suggested that the glycolysis and cAMP models might result in higher *V_A_*/*V_G_* ratios than the other three models.

### The ratio of additive genetic variance to total genetic variance

**Figure 3:**
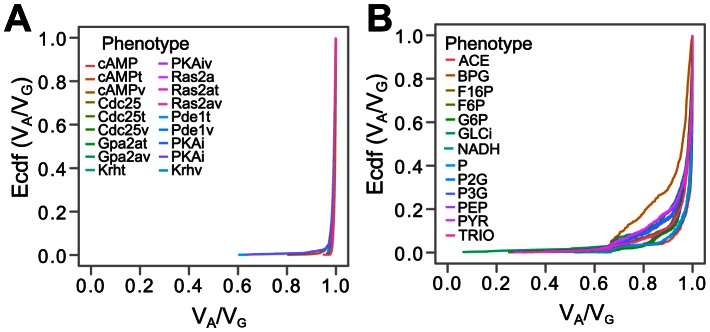
The empirical cumulative distribution function of *V_A_*/*V_G_* ratios for phenotypes of the cAMP (A) and the glycolysis (B) models. **A.** The empirical cumulative distribution functions (y axis) of *V_A_*/*V_G_* ratios (x axis) for all phenotypes studied in the cAMP model: The initial steady state concentrations before adding external glucose of the cyclic adenosine monophosphate (cAMP), the G-protein Ras2a (Ras2a), the guanine-nucleotide-exchange factor (Cdc25), the protein kinase A (PKAi). The peak values after adding glucose of these proteins (cAMPv, Ras2av, Cdc25v and PKAiv), the Kelch repeat homologue protein (Krhv), the G-protein Gpa2a (Gpa2av), and the phosphodiesterase (Pde1v). The time taken to reach the peak values (cAMPt, Ras2at, Cdc25t, PKAit, Krht, Gpa2at, Ped1t). See Table S6 for further phenotype descriptions and numerical summaries of the distribution of *V_A_*/*V_G_* ratios. **B.** The empirical cumulative distribution function (y axis) of *V_A_*/*V_G_* ratios (x axis) for the steady state concentrations of 13 metabolites in the glycolysis model: acetaldehyde (ACE), 1,3-bisphospoglycerate (BPG), fructose-1,6-bisphosphate (F16P), fructose 6-phosphate (F6P), glucose 6-phosphate (G6P), glucose in cell (GLCi), nicotinamide adenine dinucleotide (NADH), phosphates in adenine nucleotide (P), 2-phosphoglyerate (P2G), 3-phosphoglycerate (P3G), phosphoenolpyruvate (PEP), pyruvate (PYP), and trio-phosphate (TRIO). See Table S7 for further phenotype descriptions and numerical summaries of the distribution of *V_A_*/*V_G_* ratios.

The results confirmed our expectations regarding high *V_A_*/*V_G_* ratios for the glycolysis and cAMP models. Furthermore, a number of distinct patterns emerged. The cAMP model shows the overall highest *V_A_*/*V_G_* ratios values (Figure 3A and Table S6), with mean and median values above 0.99 across all recorded phenotypes. The 0.05-quantile (i.e. only 5 percent of the Monte Carlo simulations show lower values than this) *V_A_*/*V_G_* values were above 0.97 for all phenotypes and no values lower than 0.6 were observed. In other words, an intra- and inter-locus additive model of gene action very well approximates the genotype-phenotype maps arising from this cGP model.

The glycolysis model also has mean and median *V_A_*/*V_G_* values close to 1 for all phenotypes (Figure 3B and Table S7). But compared to the cAMP model, the numbers are clearly lower; the lowest recorded mean value (phenotype *BPG*) is 0.9 and 0.05-quantile values are below 0.7 for some phenotypes. A few *V_A_*/*V_G_* values below 0.5 are observed for all phenotypes. The distribution of *V_A_*/*V_G_* ratios for the cell cycle model (Figure S7 and Table S8) is quite similar to that of the glycolysis model, with a lowest mean *V_A_*/*V_G_* value of 0.93 for *time to peak* for *Sic1* and with 0.05-quantiles below 0.8 for some phenotypes. *V_A_*/*V_G_* values below 0.1 are observed for a few Monte Carlo simulations in some phenotypes.

**Figure 4:**
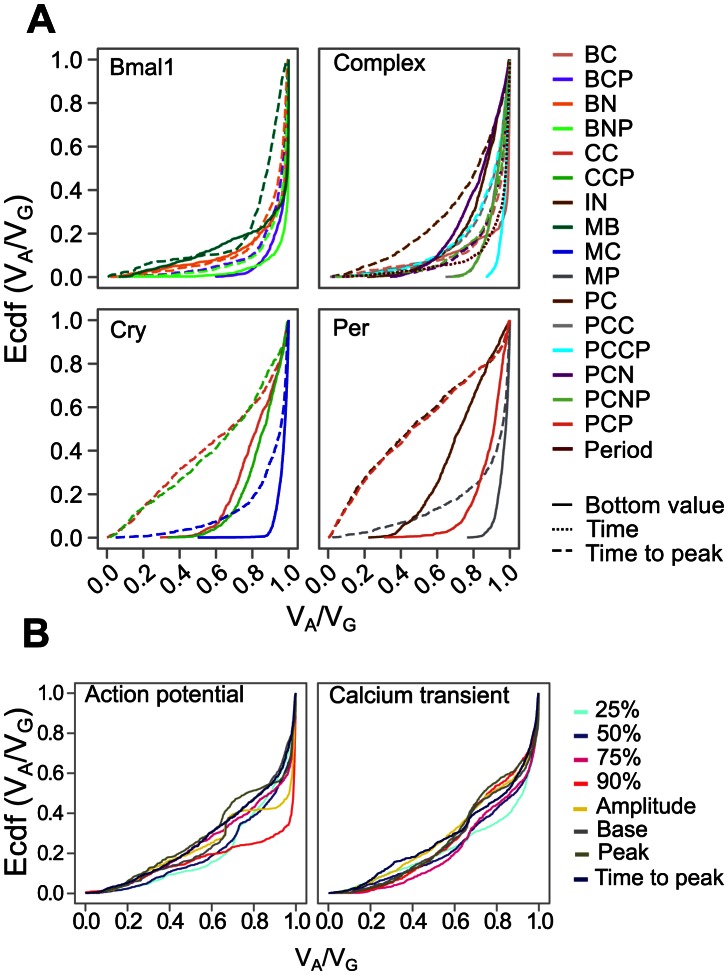
The empirical cumulative distribution function of *V_A_*/*V_G_* ratios for phenotypes of the circadian model (A) and the action potential model (B). The empirical cumulative distribution functions (y axis) of *V_A_*/*V_G_* ratios (x axis) for phenotypes studied in the circadian model and the heart cell model. **A.** The upper-left panel (Bmal1) shows phenotypes related to *bmal1* gene, including the mRNA (MB), the unphosphorylated/phosphorylated protein in cytosol (BC/BCP) and nucleus (BN/BNP). The bottom-right panel (Per) is for *per* gene, including the mRNA (MP), the unphosphorylated protein (PC) and the phosphorylated protein (PCP). The bottom concentration (solid line) and the time take to peak (dashed line) of each species are recorded phenotypes. The bottom-left panel (Cry) is related to *cry* gene, including the mRNA (MC), the unphosphorylated protein (CC) and phosphorylated protein (CCP). The upper-right panel (Complex) is for protein complexes PCC, PCN, PCCP and PCNP. The period of circadian rhythm (Period, dotted line) is also shown. See Table S9 for further phenotype descriptions and numerical summaries of the distribution of *V_A_*/*V_G_* ratios. **B.** The empirical cumulative distribution functions (y axis) of *V_A_*/*V_G_* ratios (x axis) for phenotypes studied in the action potential model: time to 25%, 50%, 75% and 90% of initial values, the amplitude, initial values (Base), peak values, time to reach peak values of action potential (left panel) and calcium transient (right panel) are shown. See Table S10 for further phenotype descriptions and numerical summaries of the distribution of *V_A_*/*V_G_* ratios.

For each of the cAMP, glycolysis and cell cycle models the distributions of *V_A_*/*V_G_* ratios were quite similar across all phenotypes, and a large majority of the Monte Carlo simulations showed very high ratios. The circadian clock model differs from these three models both in terms of displaying large variation between phenotypes and in terms of having a much larger proportion of low *V_A_*/*V_G_* values (Figure 4A and Table S9). Four phenotypes stand out with *V_A_*/*V_G_* distributions that resemble a uniform distribution *U*(0,1). These are the time from bottom to peak for the phosphorylated and unphosphorylated proteins of *Per* and *Cry*, and they have median *V_A_*/*V_G_* values ranging from 0.46 to 0.70 and 0.05-quantile values in the range 0.04 to 0.10. The remaining phenotypes have somewhat higher *V_A_*/*V_G_* values, but over half of the recorded phenotypes have 0.05-quantiles below 0.6. Median *V_A_*/*V_G_* values are below 0.9 for the majority of phenotypes of the action potential model. And all recorded phenotypes have a large proportion of low *V_A_*/*V_G_* ratios (Figure 4B and Table S10) with 0.05-quantiles in the range 0.18-0-35. The distributions are quite similar across action potential and calcium transient phenotypes, but the time to 90% repolarization for the action potential shows somewhat higher values than the others.

All five cGP models are capable of creating *V_A_*/*V_G_* ratios close to 1, and except for two phenotypes for the circadian model all median values of *V_A_*/*V_G_* are well above 0.5. This supports the hypothesis [30] that biological systems tend to involve regulatory machinery that in general leads to considerable additive genetic variance even at intermediate allele frequencies. That is not to say that selection cannot sometimes produce regulatory solutions that tend towards low *V_A_*/*V_G_* ratios; in fact, the incidence of low *V_A_*/*V_G_* ratios varied markedly among the five models that we studied. Because the genotype-parameter maps were purely additive, all non-additive genetic variance is a result of non-linearity in the ODE models. The expected effect of introducing non-additivity in the genotype-parameter maps would be a further decrease in the *V_A_*/*V_G_* ratios.

Our finding that models with complex regulation involving positive feedback loops tend to give lower *V_A_*/*V_G_* agrees with a previous study on gene regulatory networks [11]. Considering the relatively high *V_A_*/*V_G_* ratios of the cell cycle model compared to the circadian and action potential models, the following quote from Tyson and Novak's [34] discussion of why the cell-cycle is better understood as a hysteresis loop than as a limit cycle oscillator (LCO), is highly informative: “*Generally speaking, the period of an LCO is a complicated function of all the kinetic parameters in the differential equations. However, the period of the cell division cycle depends on only one kinetic property of the cell: its mass-doubling time.*” This seems to explain why the genotype-phenotype maps arising from the cell-cycle models are much more linear than the maps from the circadian model, which is an LCO.

### Monotonicity explains much of the V_A_/V_G_ patterns

In a given population *V_A_*/*V_G_* is a function of allele frequencies as well as the GP map, and GP maps with strong interactions can still give high *V_A_*/*V_G_* values in populations with extreme allele frequencies [3]. In populations with intermediate allele frequencies the *V_A_*/*V_G_* values are determined mainly by the shape of the genotype-phenotype map, and the observed differences between the five cGP models in the distribution of *V_A_*/*V_G_* values motivates a search for underlying explanatory principles.

**Figure 5:**
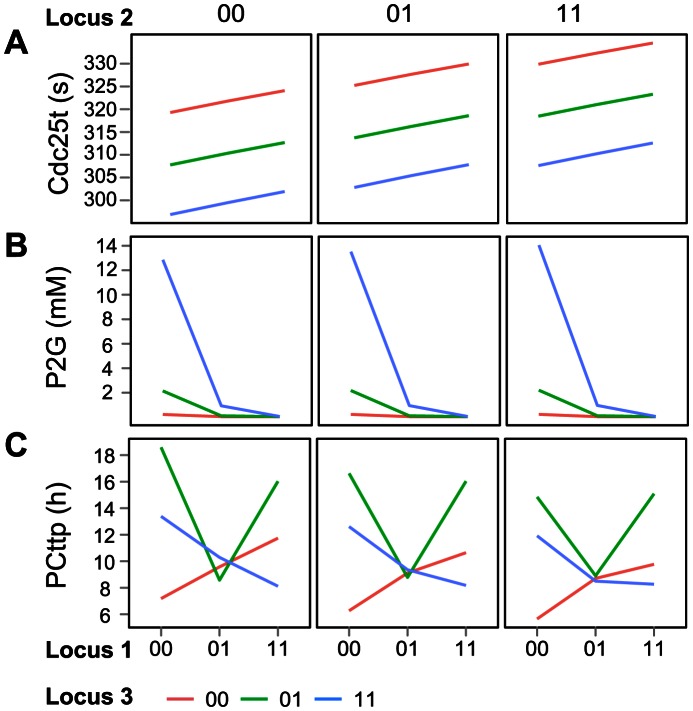
Three distinct types of genotype-phenotype maps. Examples of three distinct types of genotype-phenotype maps seen in our simulations. For each subfigure the phenotypic value is shown on the y-axis while the x-axises, line colours and plot columns indicate the genotype at the three loci. **A.** A nearly additive map exemplified by the GP map of the time to peak concentration of Cdc25 (*V_A_*/*V_G_* = 0.99) in the cAMP model; **B.** A fully monotone but non-additive map exemplified by the GP map of the concentration of P2G protein (*V_A_*/*V_G_* = 0.41) in the glycolysis model; and, **C.** A strongly non-monotonic map is found the time to peak concentration of the PC protein (*V_A_*/*V_G_* = 0.03) from the circadian model.

The recently proposed concept of monotonicity (or order-preservation) of GP maps seems to be one such principle. In short, a GP map is said to be monotone if the ordering of genotypes by gene content (the number of alleles of a given type) is preserved in the ordering of the associated phenotypic values (see [30] for details). Figure 5 depicts three extreme types of GP maps seen in our simulations. Nearly additive GP maps as shown in Figure 5A give *V_A_*/*V_G_* values very close to one. GP maps with strong magnitude epistasis, but still order-preserving, typically result in intermediate *V_A_*/*V_G_* values (Figure 5B), while highly non-monotone or order-breaking GP maps (Figure 5C) showing strong overdominance and/or sign epistasis result in *V_A_*/*V_G_* values close to zero.

**Figure 6:**
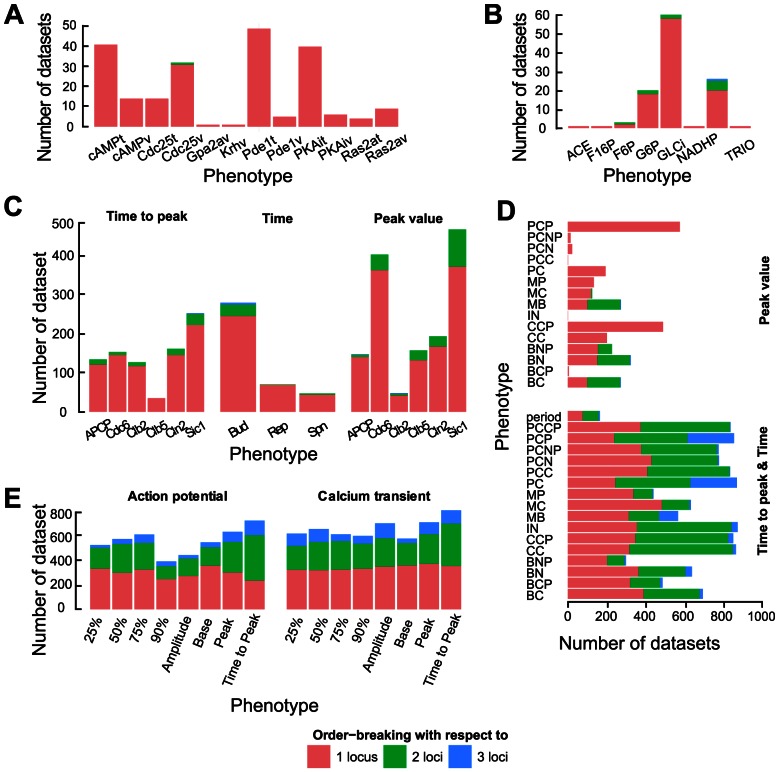
The number of loci for which the GP-map shows order-breaking. The number of Monte Carlo simulations where the GP-map for a given phenotype is clearly order-breaking (GP maps with *N/A*>0.05, see Methods) is shown for the cAMP model (**A**), the glycolysis model (**B**), the cell cycle model (**C**), the circadian model (**D**) and the action potential model (**E**). Only phenotypes with at least one Monte carlo simulation resulting in an order-breaking GP map are shown.

Based on recent results from studies of gene regulatory networks [30], we anticipated that the three cGP models with complex regulation involving positive feedback would result in considerably more non-monotone or order-breaking GP maps than the two pathway models. To test this, we measured the amount of order-breaking in all simulated GP maps (see Methods) and found a very clear pattern (Figure 6). While the datasets from the glycolysis and cAMP models contained only 1.1% and 1.3% GP maps with order-breaking for any locus, those from the cell cycle, circadian and action potential models contained 20.7%, 44.4% and 69.5%, respectively. Moreover, monotone GP maps gave higher *V_A_*/*V_G_* values than non-monotone GP maps for all five cGP models (Mann-Whitney test; p-values below 1e-10 for all five models).

However, despite the fact that the glycolysis model rarely shows order-breaking even for a single locus, it possesses much lower *V_A_*/*V_G_* values than the cAMP model. A plausible explanation is that the steady-state concentrations of metabolites can markedly increase for parameter values close to a saddle-node bifurcation point [26]. Simulation outcomes with unstable steady states were discarded, but in those cases where one of the genotypes (i.e. parameter sets) come close to the bifurcation point without crossing it we get genotype-phenotype maps as in Figure 5B, where one genotype (or a small set) gives much higher phenotypic values than the others. Such GP maps, similar to the duplicate factor model in Hill *et al.* [3], are known to give low *V_A_*/*V_G_* ratios despite being monotonic. Similar GP maps giving *V_A_*/*V_G_* ratios close to zero were also found by Keightley [32] in his study of metabolic models possessing null alleles at all loci.

### Considerations on the genericity of the results

Our main reason for restricting the sampled genetic variation of parameters to within 30% of the published baseline values was to avoid qualitative (or topological) changes of the dynamics. Such qualitative changes are often biologically realistic descriptions of knockouts or other large genetic changes, for example action potentials of alternating amplitude (alternans) [17]; loss of stable circadian oscillation [23]; and non-viable cell-cycle mutants phenotypes [22]. However, since the heritability and variance component concepts are defined for phenotypes showing continuous rather than discrete variation, we sought to avoid such qualitative changes here.

We ran simulations with five polymorphic loci for the cAMP (Figure S8A), glycolysis (Figure S8B), cell cycle (Figure S9) and action potential (Figure S10) models (the circadian model describes only three genes explicitly). The resulting *V_A_*/*V_G_* values were slightly lower than with three loci, but the overall shape of the distributions and the clear differences between models did not change. This indicates that our findings are of general relevance for oligogenic traits.

It should be emphasized that the five studied cGP models differ in several other aspects than those highlighted here, such as the system size (number of state variables) and the process time scales. These features could also contribute to the observed variation in the distributions of *V_A_*/*V_G_* ratios. However, such structural differences are unavoidable when the aim is to compare experimentally validated models designed to describe specific biological systems. A complementary approach is to study generic models where system size and equation structure is fixed, while the connectivity matrix can be changed to describe a family of systems [35]. This facilitates graph-theoretic comparison of systems at the expense of some biological realism. We anticipate that the major conclusions from such studies will be similar to ours, but it may very well be that other important generic insights may also come to the fore.

All the models in our study describe parts of the cellular machinery and the resulting phenotypes are thus cellular rather than organismal. We do not think this is a major shortcoming in terms of the main conclusions that emerge from our results. However, we anticipate that application of our approach on multiscale models including cellular, tissue and whole-organ phenotypes [36] will provide a much improved foundation for explaining how properties of the GP map vary across and within biological systems in terms of regulatory anatomy and associated genetic variation [37], [38].

As our approach can be used together with any computational biology model, it has the potential to contribute substantially to a theoretical foundation capable of predicting when we are to expect low or high *V_A_*/*V_G_* or *h^2^*/*H^2^* ratios from the principles of regulatory biology. Causally cohesive genotype-phenotype modeling thus appears to qualify as a promising approach for integrating causal models of biological networks and physiology with quantitative genetics [39]–[44].

## Supporting Information

Figure S1 **Flowchart of Monte Carlo simulations and analysis.** Flowchart of the Monte Carlo simulations described in the Methods section “Monte Carlo simulations” and subsequent analysis described in the Methods section “Statistical analysis”.
(PDF)

Figure S2 **Graphical representation of cAMP model.** Figure modified from http://www.biomedcentral.com/1752-0509/3/70/figure/F7. Red numbers, correspond to the rows in Table S1, and indicate the model elements where genetic variation was introduced.
(PDF)

Figure S3 **Graphical representation of glycolysis model.** Figure modified from the CellML model repository (http://models.cellml.org/workspace/teusink_passarge_reijenga_esgalhado_vanderweijden_schepper_walsh_bakker_vandam_westerhoff_snoep_2000). Red numbers, correspond to the rows in Table S2, and indicate the model elements where genetic variation was introduced.
(PDF)

Figure S4 **Graphical representation of cell cycle model.** Figure modified from the CellML model repository (http://models.cellml.org/workspace/chen_calzone_csikasznagy_cross_novak_tyson_2004). Red numbers, correspond to the rows in Table S3, and indicate the model elements where genetic variation was introduced.
(PDF)

Figure S5 **Graphical representation of circadian model.** Figure modified from the CellML model repository (http://models.cellml.org/workspace/leloup_goldbeter_2004). Red numbers, correspond to the rows in Table S4, and indicate the model elements where genetic variation was introduced.
(PDF)

Figure S6 **Graphical representation of action potential model.** Figure modified from the CellML model repository (http://models.cellml.org/workspace/bondarenko_szigeti_bett_kim_rasmusson_2004). Red numbers, correspond to the rows in Table S5, and indicate the model elements where genetic variation was introduced.
(PDF)

Figure S7 **The empirical cumulative distribution function of** ***V_A_*** **/** ***V_G_*** **ratios for phenotypes of the cell cycle model.** The empirical cumulative distribution functions (y axis) of *V_A_*/*V_G_* ratios (x axis) for all phenotypes studied in the cell cycle model. The phenotypes are divided into 3 groups. Cell events refer to the discrete events defined in the model paper and include timing of budding (Bud), timing of DNA replication (Rep) and timing of alignment of chromosomes on the metaphase plates (Spn). Peak concentration include the concentration of the phosphorylated anaphase-promoting complex (APCP), the G1-stabilizing protein Cdc6, the B-type Cyclin protein 2 (Clb2), the S-phase promoting B-type Cyclin (Clb5), the starter kinase (Cln2) and the G1 phase stabilizing protein (Sci1). The time to peak phenotypes include the time to reach peak concentrations of APCP, Cdc6, Clb2, Clb5, Cln2 and Sci1. See Table S8 for further phenotype descriptions and numerical summaries of the distribution of *V_A_*/*V_G_* ratios.
(PDF)

Figure S8 **The empirical cumulative distribution function of** ***V_A_*** **/** ***V_G_*** **ratios for phenotypes of the cAMP (A) and the glycolysis (B) models with 5 polymorphic loci.** Figure 3 shows results from simulations with 3 polymorhpic loci. **A.** The empirical cumulative distribution functions (y axis) of *V_A_*/*V_G_* ratios (x axis) for all phenotypes studied in the cAMP model: The initial steady state concentrations before adding external glucose of the cyclic adenosine monophosphate (cAMP), the G-protein Ras2a (Ras2a), the guanine-nucleotide-exchange factor (Cdc25), the protein kinase A (PKAi). The peak values after adding glucose of these proteins (cAMPv, Ras2av, Cdc25v and PKAiv), the Kelch repeat homologue protein (Krhv), the G-protein Gpa2a (Gpa2av), and the phosphodiesterase (Pde1v). The time taken to reach the peak values (cAMPt, Ras2at, Cdc25t, PKAit, Krht, Gpa2at, Ped1t). **B.** The empirical cumulative distribution function (y axis) of *V_A_*/*V_G_* ratios (x axis) for the steady state concentrations of 13 metabolites in the glycolysis model acetaldehyde (ACE), 1,3-bisphospoglycerate (BPG), fructose-1,6-bisphosphate (F16P), fructose 6-phosphate (F6P), glucose 6-phosphate (G6P), glucose in cell (GLCi), nicotinamide adenine dinucleotide (NADH), phosphates in adenine nucleotide (P), 2-phosphoglyerate (P2G), 3-phosphoglycerate (P3G), phosphoenolpyruvate (PEP), pyruvate (PYP), and trio-phosphate (TRIO).
(PDF)

Figure S9 **The empirical cumulative distribution function of** ***V_A_*** **/** ***V_G_*** **ratios for phenotypes of the cell cycle model with 5 polymorphic loci.** Figure S7 shows results from simulations with 3 polymorhpic loci. The empirical cumulative distribution functions (y axis) of *V_A_*/*V_G_* ratios (x axis) for all phenotypes studied in the cell cycle model. The phenotypes are divided into 3 groups. Cell events refer to the discrete events defined in the model paper and include timing of budding (Bud), timing of DNA replication (Rep) and timing of alignment of chromosomes on the metaphase plates (Spn). Peak concentration include the concentration of the phosphorylated anaphase-promoting complex (APCP), the G1-stabilizing protein Cdc6, the B-type Cyclin protein 2 (Clb2), the S-phase promoting B-type Cyclin (Clb5), the starter kinase (Cln2) and the G1 phase stabilizing protein (Sci1). The time to peak phenotypes include the time to reach peak concentrations of APCP, Cdc6, Clb2, Clb5, Cln2 and Sci1.
(PDF)

Figure S10 **The empirical cumulative distribution function of** ***V_A_*** **/** ***V_G_*** **ratios for phenotypes of the action potential model with 5 polymorphic loci.** Figure 4B shows results from simulations with 3 polymorhpic loci. The empirical cumulative distribution functions (y axis) of *V_A_*/*V_G_* ratios (x axis) for phenotypes studied in the action potential model: time to 25%, 50%, 75% and 90% of initial values, the amplitude, initial values (Base), peak values, time to reach peak values of action potential (left panel) and calcium transient (right panel) are shown.
(PDF)

Table S1 **Polymorphic model elements of the cAMP model.** A list of cAMP model elements and parameters used to manifest genetic variation. Parameter names from the original publication (Table 4 in [21], names used in the SBML file retrieved from http://www.biomedcentral.com/content/supplementary/1752-0509-3-70-s1.xml and baseline values with units.
(PDF)

Table S2 **Polymorphic model elements of the glycolysis model.** A list of glycolysis model elements and parameters used to manifest genetic variation. Parameter names from the original publication [20], names used in the SBML file retrieved from http://www.ebi.ac.uk/biomodels-main/BIOMD0000000064 and baseline values with units.
(PDF)

Table S3 **Polymorphic model elements of the cell cycle model.** A list of cell cycle model elements and parameters used to manifest genetic variation. Parameter names from Table 1 and Table 2 in the original publication [22], names used in the CellML file retrieved from http://models.cellml.org/workspace/chen_calzone_csikasznagy_cross_novak_tyson_2004 and baseline values with units.
(PDF)

Table S4 **Polymorphic model elements of the circadian model.** A list of circadian model elements and parameters used to manifest genetic variation. Parameter names from Table 1 (parameter set 4) in the original publication [23], names used in the CellML file “leloup_goldbeter_2004.cellml” retrieved from http://models.cellml.org/workspace/leloup_goldbeter_2004/ and baseline values with units.
(PDF)

Table S5 **Polymorphic model elements of the action potential model.** A list of action potential model elements and parameters used to manifest genetic variation. Parameter names from Table B1 in the original publication [24], names used in the CellML file which is available as supplementary material (filename “LNCS model.zip”) at doi:10.3389/fphys.2011.00106 and baseline values with units.
(PDF)

Table S6 **Summary of phenotype descriptions, variability thresholds and distribution of** ***V_A_*** **/** ***V_G_*** **ratios for the cAMP model.** The first three columns list the phenotype abbreviations used in this study, a text description of the phenotypes and their units. The thresholds used to filter out dataset with very low relative and/or absolute variability are listed in the next two columns, followed by the number of Monte Carlo simulations (out of 1000) passing the threshold. The last 7 columns contain quantiles and means of the *V_A_*/*V_G_* values for the datasets passing the variability threshold.
(PDF)

Table S7 **Summary of phenotype descriptions, variability thresholds and distribution of** ***V_A_*** **/** ***V_G_*** **ratios for the glycolysis model.** The first three columns list the phenotype abbreviations used in this study, a text description of the phenotypes and their units. The thresholds used to filter out dataset with very low relative and/or absolute variability are listed in the next two columns, followed by the number of Monte Carlo simulations (out of 1000) passing the threshold. The last 7 columns contain quantiles and means of the *V_A_*/*V_G_* values for the datasets passing the variability threshold.
(PDF)

Table S8 **Summary of phenotype descriptions, variability thresholds and distribution of** ***V_A_*** **/** ***V_G_*** **ratios for the cell cycle model.** The first three columns list the phenotype abbreviations used in this study, a text description of the phenotypes and their units. The thresholds used to filter out dataset with very low relative and/or absolute variability are listed in the next two columns, followed by the number of Monte Carlo simulations (out of 1000) passing the threshold. The last 7 columns contain quantiles and means of the *V_A_*/*V_G_* values for the datasets passing the variability threshold.
(PDF)

Table S9 **Summary of phenotype descriptions, variability thresholds and distribution of** ***V_A_*** **/** ***V_G_*** **ratios for the circadian model.** The first three columns list the phenotype abbreviations used in this study, a text description of the phenotypes and their units. The thresholds used to filter out dataset with very low relative and/or absolute variability are listed in the next two columns, followed by the number of Monte Carlo simulations (out of 1000) passing the threshold. The last 7 columns contain quantiles and means of the *V_A_*/*V_G_* values for the datasets passing the variability threshold. Abbreviations: phosphorylated – phos., cytosolic – cyt., nuclear – nuc., bottom concentration – b.c., peak concentration – p.c.
(PDF)

Table S10 **Summary of phenotype descriptions, variability thresholds and distribution of** ***V_A_*** **/** ***V_G_*** **ratios for the action potential model.** The first three columns list the phenotype abbreviations used in this study, a text description of the phenotypes and their units. The thresholds used to filter out dataset with very low relative and/or absolute variability are listed in the next two columns, followed by the number of Monte Carlo simulations (out of 1000) passing the threshold. The last 7 columns contain quantiles and means of the *V_A_*/*V_G_* values for the datasets passing the variability threshold.
(PDF)

Text S1 **More detailed descriptions of the five cGP models.**
(PDF)
