# Supplementary figures and images for: Effect of Regulatory Architecture on Broad versus Narrow Sense Heritability
Source: PLoS Comput Biol. 2013 May 9;9(5):e1003053. doi: 10.1371/journal.pcbi.1003053 (PMC3649986; doi:10.1371/journal.pcbi.1003053)

## Monte Carlo simulations

## Data analysis

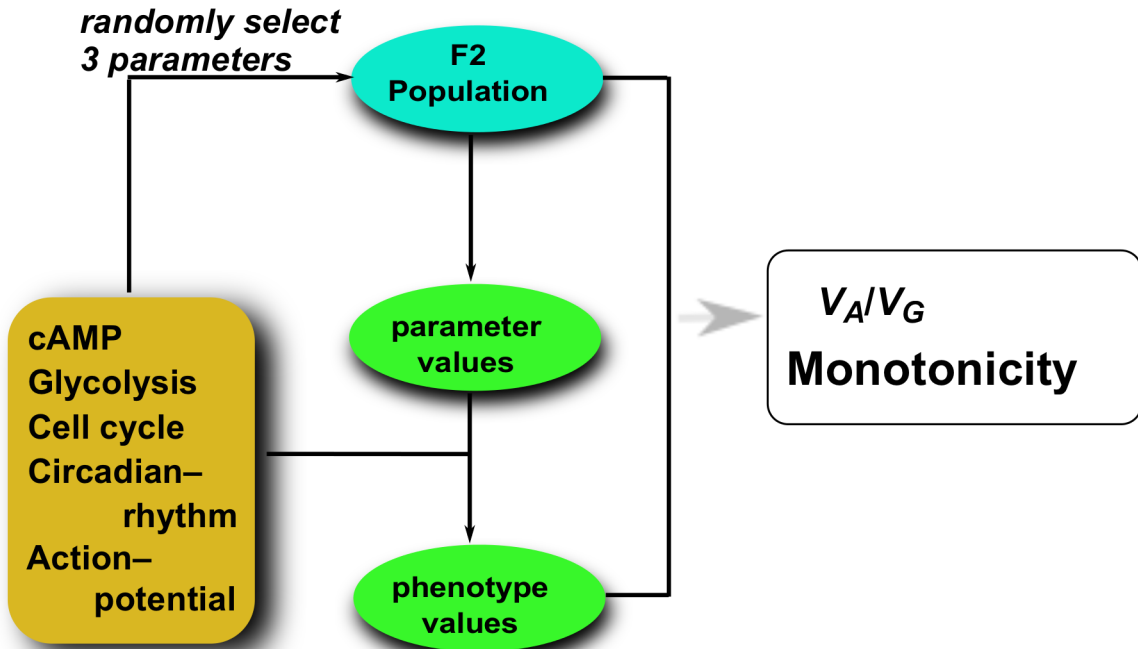

Supplement: Figure S1 — Flowchart of Monte Carlo simulations and analysis. Flowchart of the Monte Carlo simulations described in the Methods section “Monte Carlo simulations” and subsequent analysis described in the Methods section “Statistical analysis”. (PDF) [file pcbi.1003053.s001.pdf]

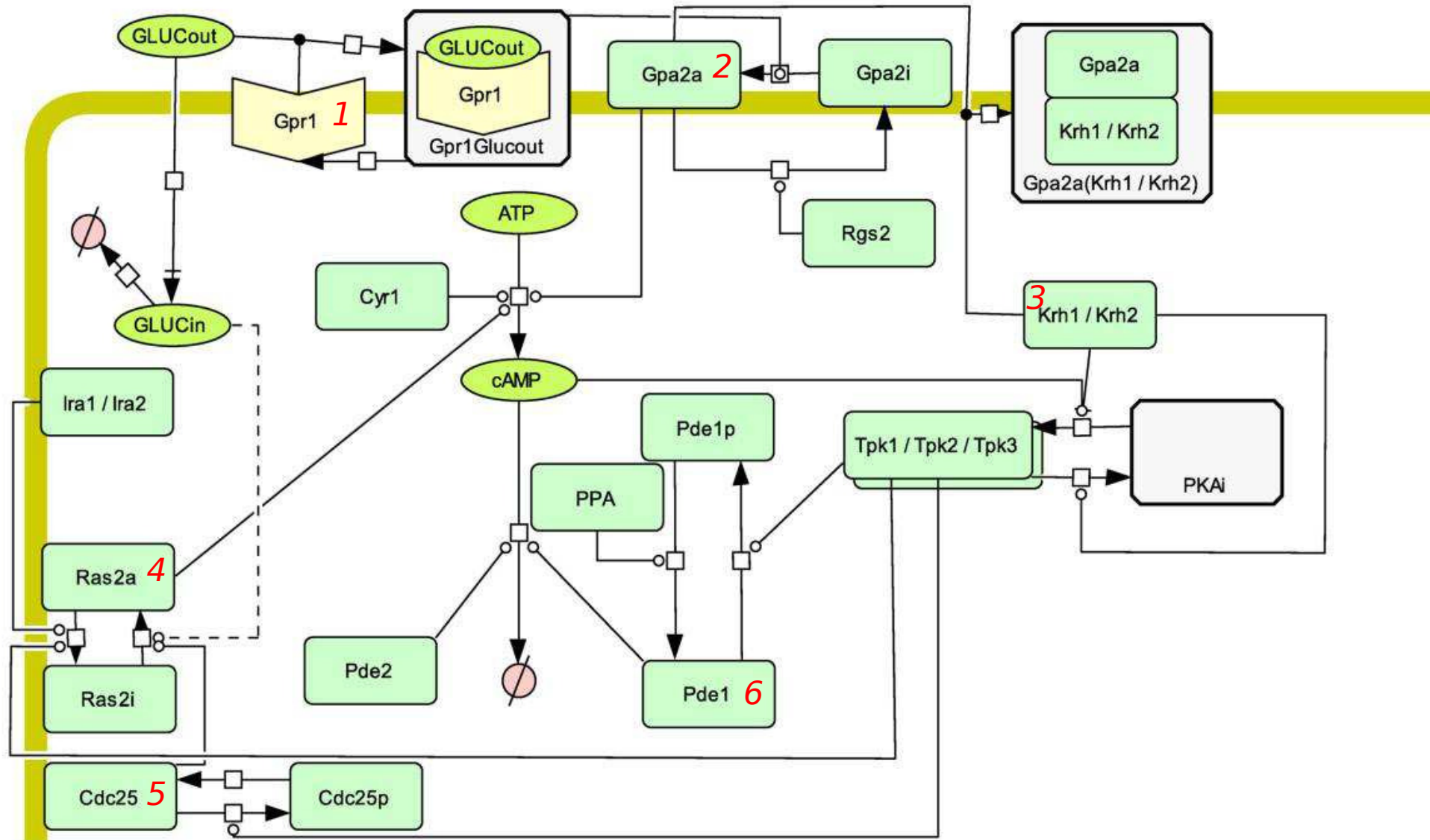

Supplement: Figure S2 — Graphical representation of cAMP model. Figure modified from http://www.biomedcentral.com/1752-0509/3/70/figure/F7. Red numbers, correspond to the rows in Table S1, and indicate the model elements where genetic variation was introduced. (PDF) [file pcbi.1003053.s002.pdf]

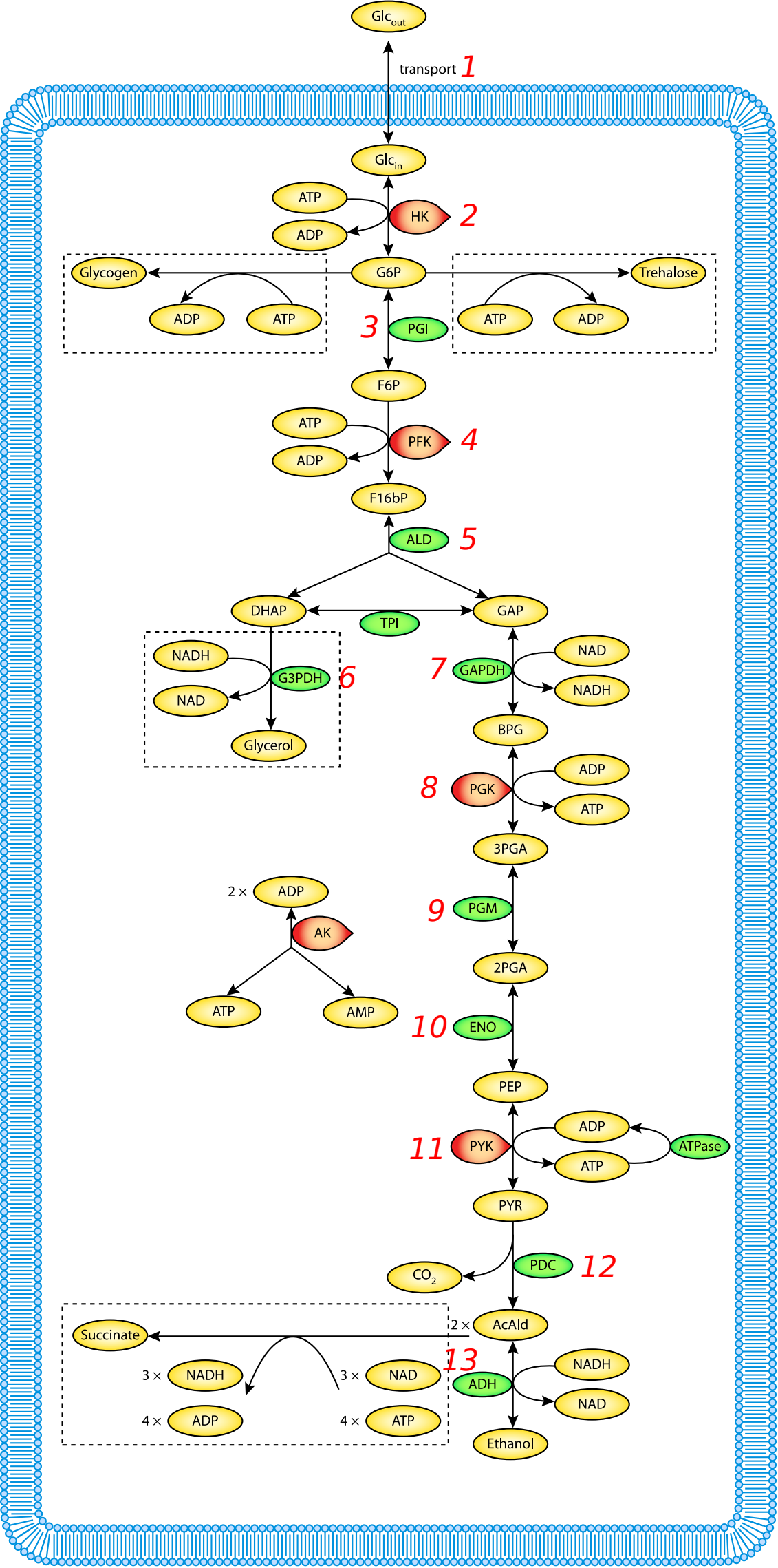

Supplement: Figure S3 — Graphical representation of glycolysis model. Figure modified from the CellML model repository (http://models.cellml.org/workspace/teusink_passarge_reijenga_esgalhado_vanderweijden_schepper_walsh_bakker_vandam_westerhoff_snoep_2000). Red numbers, correspond to the rows in Table S2, and indicate the model elements where genetic variation was introduced. (PDF) [file pcbi.1003053.s003.pdf]

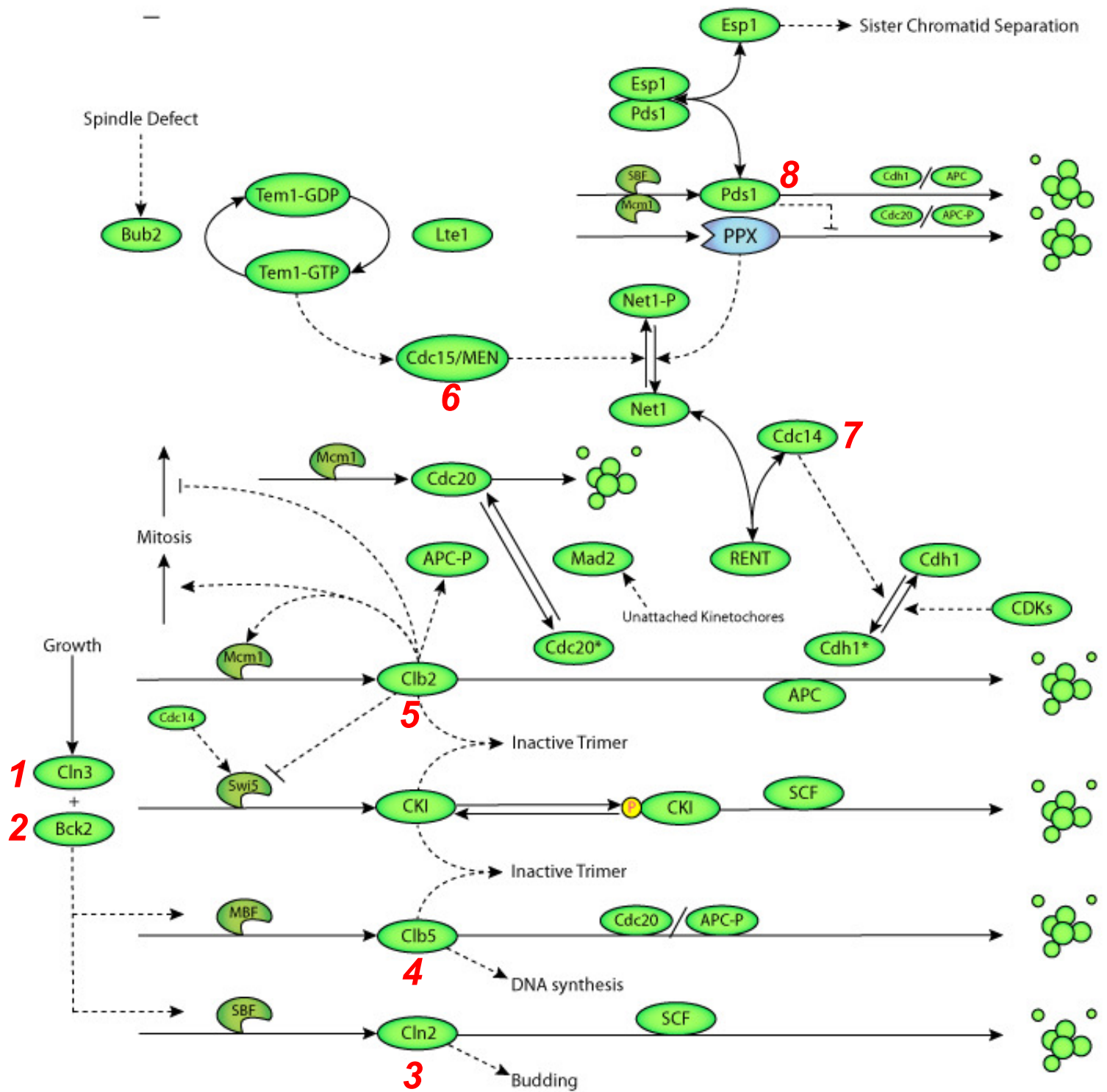

Supplement: Figure S4 — Graphical representation of cell cycle model. Figure modified from the CellML model repository (http://models.cellml.org/workspace/chen_calzone_csikasznagy_cross_novak_tyson_2004). Red numbers, correspond to the rows in Table S3, and indicate the model elements where genetic variation was introduced. (PDF) [file pcbi.1003053.s004.pdf]

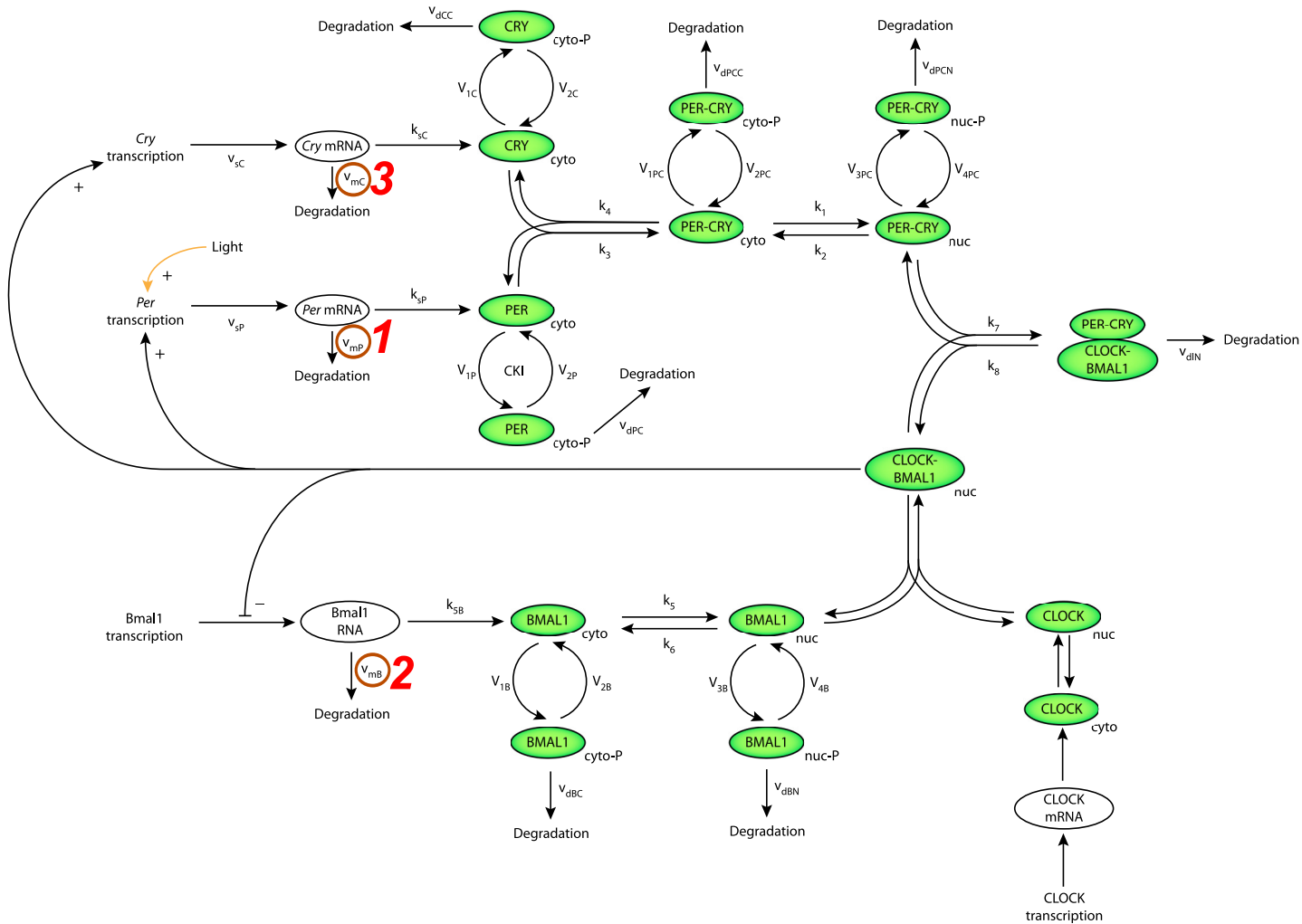

Supplement: Figure S5 — Graphical representation of circadian model. Figure modified from the CellML model repository (http://models.cellml.org/workspace/leloup_goldbeter_2004). Red numbers, correspond to the rows in Table S4, and indicate the model elements where genetic variation was introduced. (PDF) [file pcbi.1003053.s005.pdf]

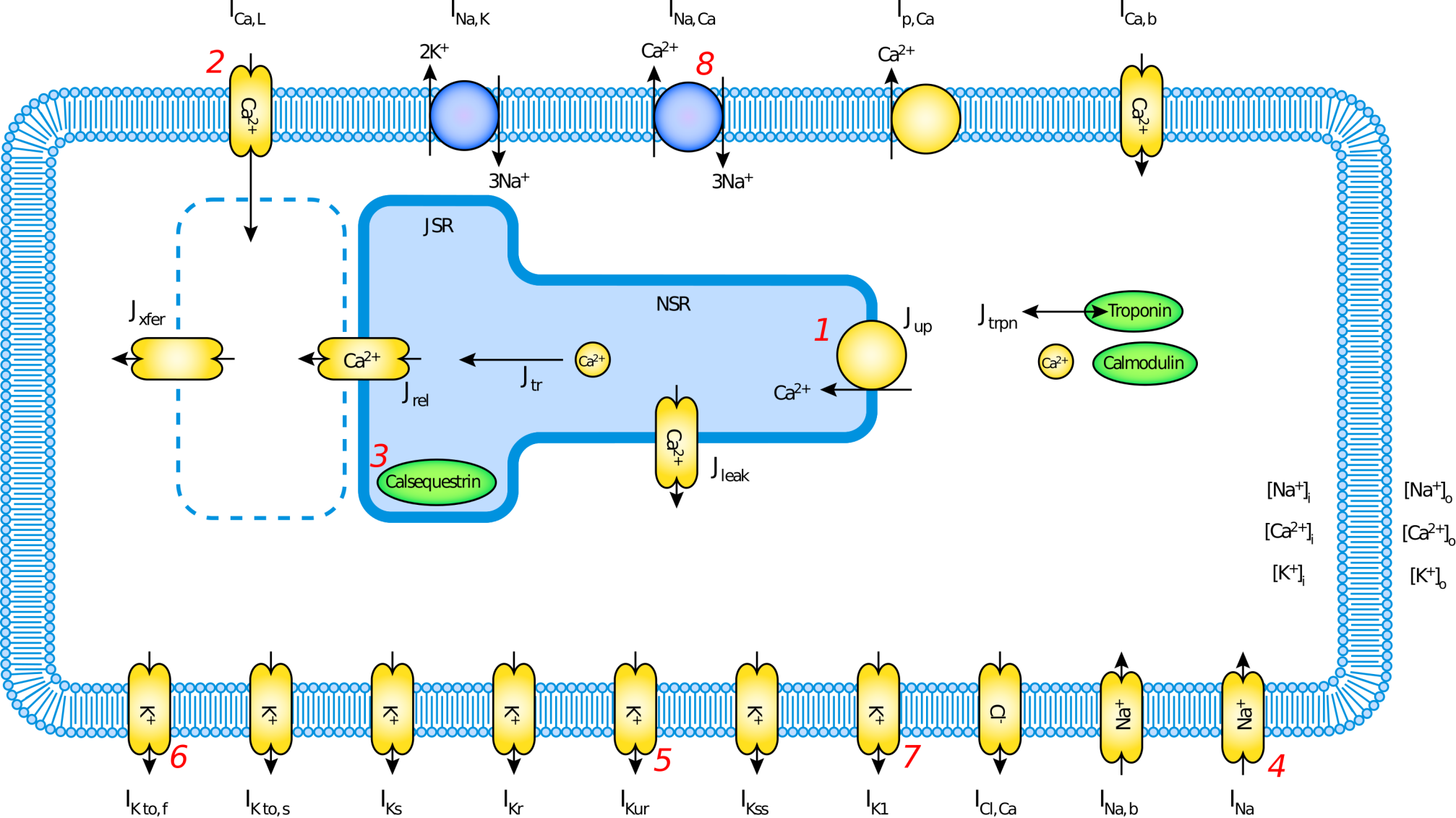

Supplement: Figure S6 — Graphical representation of action potential model. Figure modified from the CellML model repository (http://models.cellml.org/workspace/bondarenko_szigeti_bett_kim_rasmusson_2004). Red numbers, correspond to the rows in Table S5, and indicate the model elements where genetic variation was introduced. (PDF) [file pcbi.1003053.s006.pdf]

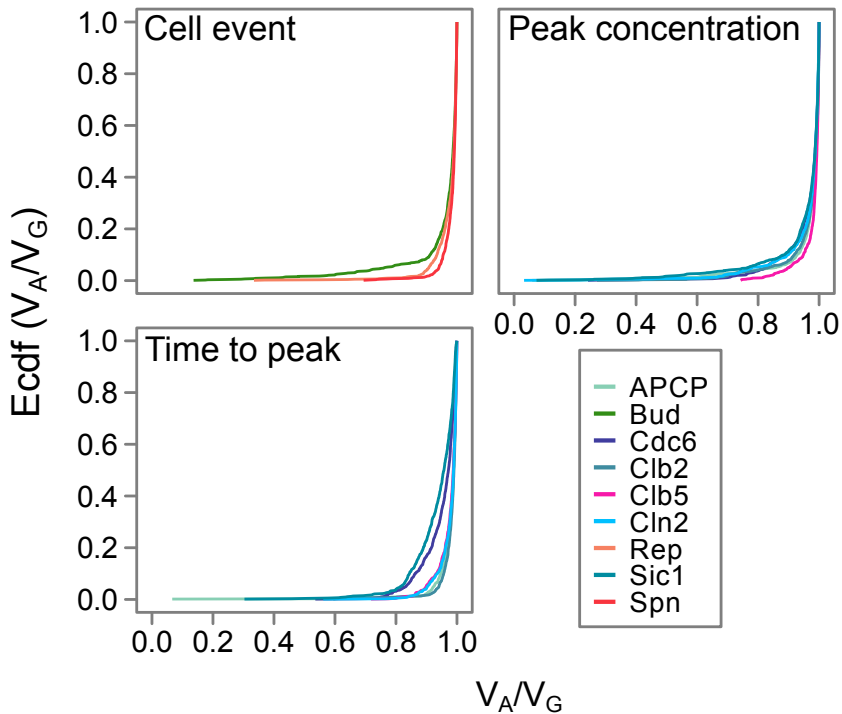

Supplement: Figure S7 — The empirical cumulative distribution function of VA / VG ratios for phenotypes of the cell cycle model. The empirical cumulative distribution functions (y axis) of VA/VG ratios (x axis) for all phenotypes studied in the cell cycle model. The phenotypes are divided into 3 groups. Cell events refer to the discrete events defined in the model paper and include timing of budding (Bud), timing of DNA replication (Rep) and timing of alignment of chromosomes on the metaphase plates (Spn). Peak concentration include the concentration of the phosphorylated anaphase-promoting complex (APCP), the G1-stabilizing protein Cdc6, the B-type Cyclin protein 2 (Clb2), the S-phase promoting B-type Cyclin (Clb5), the starter kinase (Cln2) and the G1 phase stabilizing protein (Sci1). The time to peak phenotypes include the time to reach peak concentrations of APCP, Cdc6, Clb2, Clb5, Cln2 and Sci1. See Table S8 for further phenotype descriptions and numerical summaries of the distribution of VA/VG ratios. (PDF) [file pcbi.1003053.s007.pdf]

**A**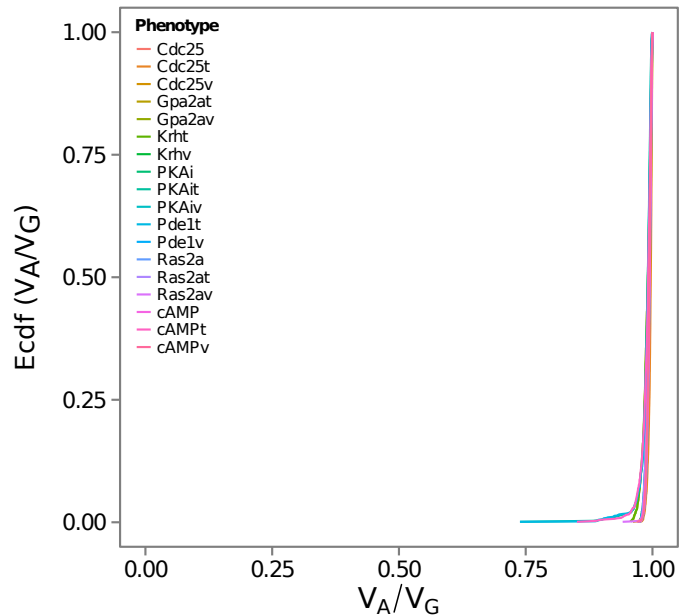**B**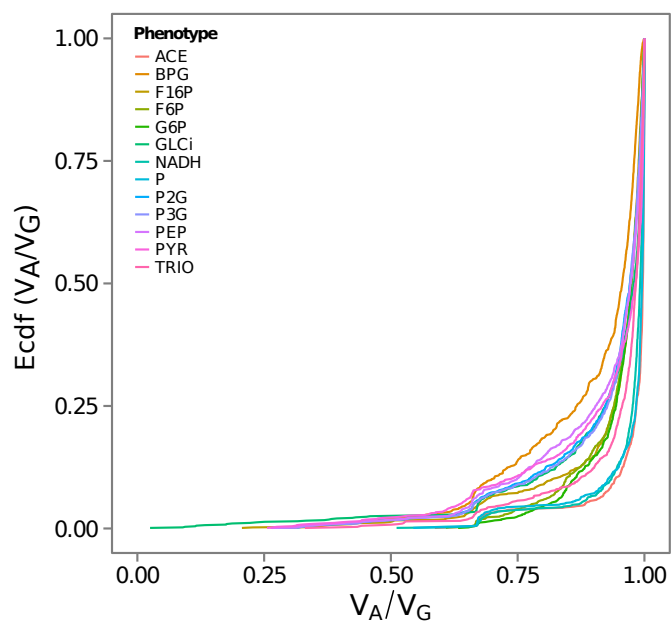

Supplement: Figure S8 — The empirical cumulative distribution function of VA / VG ratios for phenotypes of the cAMP (A) and the glycolysis (B) models with 5 polymorphic loci. Figure 3 shows results from simulations with 3 polymorhpic loci. A. The empirical cumulative distribution functions (y axis) of VA/VG ratios (x axis) for all phenotypes studied in the cAMP model: The initial steady state concentrations before adding external glucose of the cyclic adenosine monophosphate (cAMP), the G-protein Ras2a (Ras2a), the guanine-nucleotide-exchange factor (Cdc25), the protein kinase A (PKAi). The peak values after adding glucose of these proteins (cAMPv, Ras2av, Cdc25v and PKAiv), the Kelch repeat homologue protein (Krhv), the G-protein Gpa2a (Gpa2av), and the phosphodiesterase (Pde1v). The time taken to reach the peak values (cAMPt, Ras2at, Cdc25t, PKAit, Krht, Gpa2at, Ped1t). B. The empirical cumulative distribution function (y axis) of VA/VG ratios (x axis) for the steady state concentrations of 13 metabolites in the glycolysis model acetaldehyde (ACE), 1,3-bisphospoglycerate (BPG), fructose-1,6-bisphosphate (F16P), fructose 6-phosphate (F6P), glucose 6-phosphate (G6P), glucose in cell (GLCi), nicotinamide adenine dinucleotide (NADH), phosphates in adenine nucleotide (P), 2-phosphoglyerate (P2G), 3-phosphoglycerate (P3G), phosphoenolpyruvate (PEP), pyruvate (PYP), and trio-phosphate (TRIO). (PDF) [file pcbi.1003053.s008.pdf]

Ecdf ( $V_A/V_G$ )

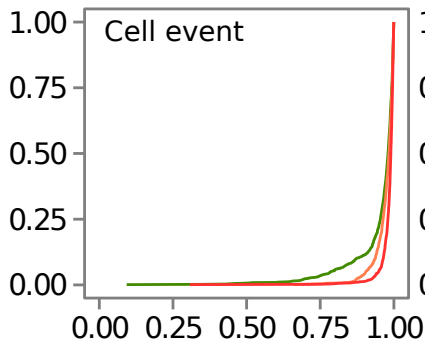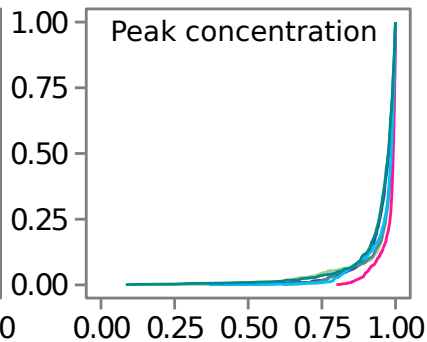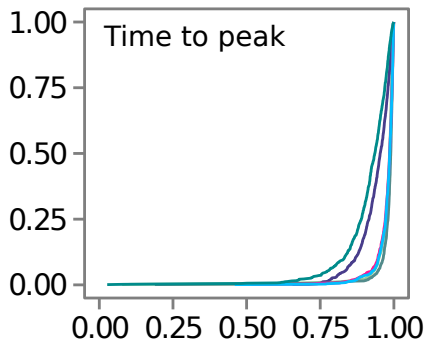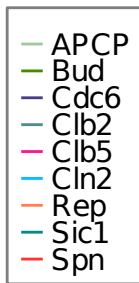

$V_A/V_G$

Supplement: Figure S9 — The empirical cumulative distribution function of VA / VG ratios for phenotypes of the cell cycle model with 5 polymorphic loci. Figure S7 shows results from simulations with 3 polymorhpic loci. The empirical cumulative distribution functions (y axis) of VA/VG ratios (x axis) for all phenotypes studied in the cell cycle model. The phenotypes are divided into 3 groups. Cell events refer to the discrete events defined in the model paper and include timing of budding (Bud), timing of DNA replication (Rep) and timing of alignment of chromosomes on the metaphase plates (Spn). Peak concentration include the concentration of the phosphorylated anaphase-promoting complex (APCP), the G1-stabilizing protein Cdc6, the B-type Cyclin protein 2 (Clb2), the S-phase promoting B-type Cyclin (Clb5), the starter kinase (Cln2) and the G1 phase stabilizing protein (Sci1). The time to peak phenotypes include the time to reach peak concentrations of APCP, Cdc6, Clb2, Clb5, Cln2 and Sci1. (PDF) [file pcbi.1003053.s009.pdf]

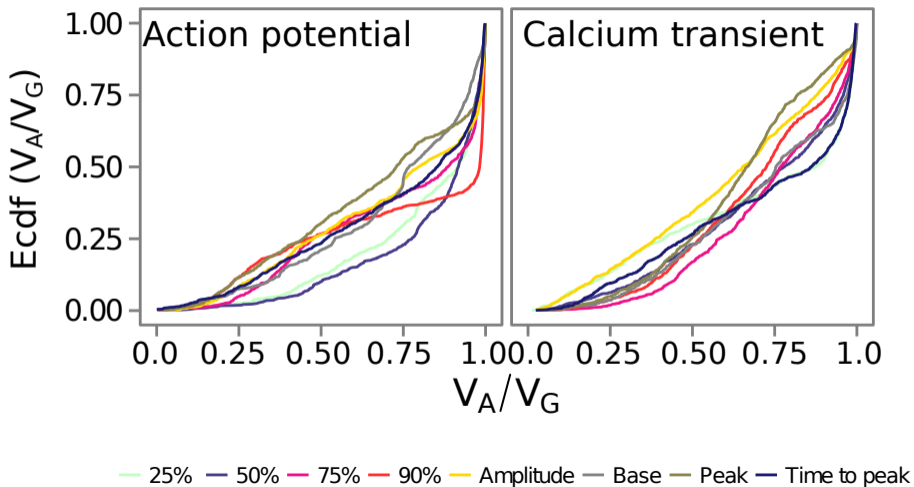

Supplement: Figure S10 — The empirical cumulative distribution function of VA / VG ratios for phenotypes of the action potential model with 5 polymorphic loci. Figure 4B shows results from simulations with 3 polymorhpic loci. The empirical cumulative distribution functions (y axis) of VA/VG ratios (x axis) for phenotypes studied in the action potential model: time to 25%, 50%, 75% and 90% of initial values, the amplitude, initial values (Base), peak values, time to reach peak values of action potential (left panel) and calcium transient (right panel) are shown. (PDF) [file pcbi.1003053.s010.pdf]
