# Supplementary material for: Effect of Regulatory Architecture on Broad versus Narrow Sense Heritability
Source: PLoS Comput Biol. 2013 May 9;9(5):e1003053. doi: 10.1371/journal.pcbi.1003053 (PMC3649986; doi:10.1371/journal.pcbi.1003053)
Supplement: Table S1 — Polymorphic model elements of the cAMP model. A list of cAMP model elements and parameters used to manifest genetic variation. Parameter names from the original publication (Table 4 in [21], names used in the SBML file retrieved from http://www.biomedcentral.com/content/supplementary/1752-0509-3-70-s1.xml and baseline values with units. (PDF) [file pcbi.1003053.s011.pdf]

Table S1. Polymorphic model elements of the cAMP model. A list of cAMP model elements and parameters used to manifest genetic variation. Parameter names from the original publication (Table 4 in [21]), names used in the SBML file retrieved from http://www.biomedcentral.com/content/supplementary/1752-0509-3-70-s1.xml and baseline values with units.

Model element	Parameter	Name in SBML file	Baseline values
G-protein coupled glucose receptor Gpr1	Gpr1 Glucose association k_1	Gpr1Glucass_k_1	0.003 L^2/mmol*s
	Gpr1 Glocuse dissociation k_1	Gpr1Glucdiss_k_2	0.14 L/s
Kelch Repeat Homologue protein Krh	Gpa2-Krh association k_F	Gpa2Krhass_k_5	391089.6 L^2/mmol*s
	Gpa2-Krh dissociation k_F	Gpa2Krhdiss_k_6	6.12 L/s
G-protein Gpa2	Gpa2 activation k_A	Gpa2act_kA_3	57682.62 L^2/mmol*s
	Gpa2 deactivation k_F	Gpa2deact_k1_4	0.899 L/s
G-protein Ras2	Ras2 activation k_{cat}	Ras2act_kcat_7	0.74 L/s
	Ras2 deactivation k_F	Ras2deact_k1_8	0.042 L/s
Guanine-nucleotide-exchange r factor Cdc25	Cdc25 phosphorylation k_{cat}	Cdc25phos_kcat_9	0.18 L/s
	Cdc25 dephosphorylation k_{cat}	Cdc25dephos_kcat_10	2.52 L/s
Phosphodiesterase Pde1	Pde1 phosphorylation k_{cat}	Pde1phos_kcat_15	6.82 L/s
	Pde1 dephosphorylation k_{cat}	Pde1dephos_kcat_16	2.4 L/s
