# Supplementary material for: Effect of Regulatory Architecture on Broad versus Narrow Sense Heritability
Source: PLoS Comput Biol. 2013 May 9;9(5):e1003053. doi: 10.1371/journal.pcbi.1003053 (PMC3649986; doi:10.1371/journal.pcbi.1003053)
Supplement: Table S2 — Polymorphic model elements of the glycolysis model. A list of glycolysis model elements and parameters used to manifest genetic variation. Parameter names from the original publication [20], names used in the SBML file retrieved from http://www.ebi.ac.uk/biomodels-main/BIOMD0000000064 and baseline values with units. (PDF) [file pcbi.1003053.s012.pdf]

Table S2. Polymorphic model elements of the glycolysis model. A list of glycolysis model elements and parameters used to manifest genetic variation. Parameter names from the original publication ([20]), names used in the SBML file retrieved from http://www.ebi.ac.uk/biomodels-main/BIOMD0000000064 and baseline values with units.

Model element	Parameter	Name in SBML file	Baseline value (SBML file)
Glucose transporter	V_{max}	vGLT_VmGLT	97.26 mmol/min
HK (Hexokinase)	V_{max}	vGLK_VmGLK	226.45 mM/min
PGI (Glucose-6-phosphate isomerase)	V_{max}	vPGI_VmPGI_2 339.68	339.68 mM/min
PFK (Phosphofructokinase)	V_{max}	vPFK_VmPFK	182.90 mM/min
ALD (Aldolase)	V_{max}	vALD_VmALD	322.26 mM/min
G3PDH (Glyceral 3-phosphate dehydrogenase)	V_{max}	vG3PDH_VmG3PDH	70.15 mM/min
GAPDH (Glyceraldehyde 3-phosphate dehydrogenase)	V_{max}	vGAPDH_VmGAPD	1184.52 mM/min
PGK (Phosphoglycerate kinase)	V_{max}	vPGK_VmPGK	1306.45 mM/min
PGM (Phosphoglycerate mutase)	V_{max}	vPGM_VmPGM	2525.81 mM/min
ENO (Enolase)	V_{max}	vENO_VmENO	365.81 mM/min
PYK (Pyruvate kinase)	V_{max}	vPYK_VmPYK	1088.71 mM/min
PDC (Pyruvate decarboxylase)	V_{max}	vPDC_VmPDC	174.19 mM/min
ADH (Alcohol dehydrogenase)	V_{max}	vADH_VmADH	810.00 mM/min
