# Supplementary material for: Effect of Regulatory Architecture on Broad versus Narrow Sense Heritability
Source: PLoS Comput Biol. 2013 May 9;9(5):e1003053. doi: 10.1371/journal.pcbi.1003053 (PMC3649986; doi:10.1371/journal.pcbi.1003053)
Supplement: Table S3 — Polymorphic model elements of the cell cycle model. A list of cell cycle model elements and parameters used to manifest genetic variation. Parameter names from Table 1 and Table 2 in the original publication [22], names used in the CellML file retrieved from http://models.cellml.org/workspace/chen_calzone_csikasznagy_cross_novak_tyson_2004 and baseline values with units. (PDF) [file pcbi.1003053.s013.pdf]

Table S3. Polymorphic model elements of the cell cycle model [22]**.** A list of cell cycle model elements and parameters used to manifest genetic variation. Parameter names from Table 1 and Table 2 in the original publication ([22]), names used in the CellML file retrieved from http://models.cellml.org/workspace/chen_calzone_csikasznagy_cross_novak_tyson_2004 and baseline values with units.

Model species	Parameters	Name in CellML file	Baseline values
Cln3	C_0	C0	0.4 dimensionless
Bck2	B_0	B0	0.054 dimensionless
Cln2	$k''_{s,n2}$	ks_n2__	0.15 min^{-1}
	$k_{d,n2}$	kd_n2	0.12 min^{-1}
Clb5	$k''_{s,b5}$	ks_b5__	0.005 min^{-1}
	$k'_{d,b5}$	kd_b5_	0.01 min^{-1}
Clb2	$k''_{s,b2}$	ks_b2__	0.04 min^{-1}
	$k'_{d,b2}$	kd_b2_	0.003 min^{-1}
Cdc15	$k''_{a,15}$	ka_15__	1 min^{-1}
	$k_{i,15}$	ki_15	0.5 min^{-1}
Cdc14	$k''_{s,14}$	ks_14	0.2 min^{-1}
	$k_{d,15}$	kd_14	0.1 min^{-1}
Pds1	$k''_{s1,pds}$	ks1_pds__	0.03 min^{-1}
	$k'_{d1,pds}$	kd1_pds_	0.01 min^{-1}
