# Supplementary material for: Effect of Regulatory Architecture on Broad versus Narrow Sense Heritability
Source: PLoS Comput Biol. 2013 May 9;9(5):e1003053. doi: 10.1371/journal.pcbi.1003053 (PMC3649986; doi:10.1371/journal.pcbi.1003053)
Supplement: Table S4 — Polymorphic model elements of the circadian model. A list of circadian model elements and parameters used to manifest genetic variation. Parameter names from Table 1 (parameter set 4) in the original publication [23], names used in the CellML file “leloup_goldbeter_2004.cellml” retrieved from http://models.cellml.org/workspace/leloup_goldbeter_2004/ and baseline values with units. (PDF) [file pcbi.1003053.s014.pdf]

Table S4. Polymorphic model elements of the circadian model [23]**.** A list of circadian model elements and parameters used to manifest genetic variation. Parameter names from Table 1 (parameter set 4) in the original publication ([23]), names used in the CellML file "leloup_goldbeter_2004.cellml" retrieved from http://models.cellml.org/workspace/leloup_goldbeter_2004/ and baseline values with units.

Model element	Parameters	Name in CellML file	Baseline values
Per	Max. rate of *Per* mRNA degradation v_{mP}	vmP	1.1 nM/h
Bmal1	Max. rate of *Bmal1* mRNA degradation v_{mB}	vmB	0.2 nM/h
Cry	Max. rate of *Cry* mRNA degradation v_{mC}	vmC	1.0 nM/h
