# Supplementary material for: Effect of Regulatory Architecture on Broad versus Narrow Sense Heritability
Source: PLoS Comput Biol. 2013 May 9;9(5):e1003053. doi: 10.1371/journal.pcbi.1003053 (PMC3649986; doi:10.1371/journal.pcbi.1003053)
Supplement: Table S5 — Polymorphic model elements of the action potential model. A list of action potential model elements and parameters used to manifest genetic variation. Parameter names from Table B1 in the original publication [24], names used in the CellML file which is available as supplementary material (filename “LNCS model.zip”) at doi:10.3389/fphys.2011.00106 and baseline values with units. (PDF) [file pcbi.1003053.s015.pdf]

Table S5. Polymorphic model elements of the action potential model. A list of action potential model elements and parameters used to manifest genetic variation. Parameter names from Table B1 in the original publication ([24]), names used in the CellML file which is available as supplementary material (filename "LNCS model.zip") at doi:10.3389/fphys.2011.00106_ and baseline values with units.

Model element	Parameters	Name in CellML file	Standard values
SERCA	$K_{m,up}$ affinity to intracellular Ca^{2+}	Km_up	0.412 uM
L-type Ca^{2+} channel	P_{CaL} permeability of the channel	P_CaL	2.5 ms^{-1}
Calsequestrin (CSQN)	K_m^{CSQN} CSQN affinity to Ca^{2+}	Km_CSQN	630 uM
Na^+ channel	G_{Na} maximum conductance	g_Na	16 mS/uF
Ultrarapidly activating delayed rectifier K^+ channel	G_{kur} : maximum conductance	g_Kur	0.25 mS/uF
Rapidly recovering transient outward K^+ channel	$G_{kto,f}$: maximum conductance	g_Kto_f	0.535 mS/uF
Time-dependent K^+ channel	maximal conductance	g_K1	0.35 mS/uF
Sodium calcium exchanger	$K_{m,Nai}$ affinity of the to intracellular Na^+	K_mNai	12 mM
