# Supplementary material for: Effect of Regulatory Architecture on Broad versus Narrow Sense Heritability
Source: PLoS Comput Biol. 2013 May 9;9(5):e1003053. doi: 10.1371/journal.pcbi.1003053 (PMC3649986; doi:10.1371/journal.pcbi.1003053)
Supplement: Table S6 — Summary of phenotype descriptions, variability thresholds and distribution of VA / VG ratios for the cAMP model. The first three columns list the phenotype abbreviations used in this study, a text description of the phenotypes and their units. The thresholds used to filter out dataset with very low relative and/or absolute variability are listed in the next two columns, followed by the number of Monte Carlo simulations (out of 1000) passing the threshold. The last 7 columns contain quantiles and means of the VA/VG values for the datasets passing the variability threshold. (PDF) [file pcbi.1003053.s016.pdf]

Table S6. Summary of phenotypic values, variability thresholds and distribution of V_A/V_G ratios for the cAMP model [21] **.** The first three columns list the phenotype abbreviations used in this study, a text description of the phenotypes and their units. The thresholds used to filter out datase with very low relative and/or absolute variability are listed in the next two columns, followed by the number of Monte Carlo simulations (out of 1000) passing the threshold. The last 7 columns contain quantiles and means of the V_A/V_G values for the datasets passing the variability threshold.

**Phenotype**	**Description**	**Units**	**Variability threshold**		**# of valid datasets**	**Quantiles and mean values of V_A/V_G**						
			**rel.**	**abs.**		**$Q_{0.05}$**	**$Q_{0.1}$**	**$Q_{0.2}$**	**$Q_{0.3}$**	**$Q_{0.5}$**	**$Q_{0.8}$**	**mean**
**cAMP**	cAMP steady state concentration (*SSC*) before adding glucose	mM	0.01	1e-8	866	0.98	0.99	0.99	0.99	0.99	1	0.99
**cAMPt**	Time to peak concentration (*PC*) of cAMP	Seconds	0.01	1	879	0.97	0.98	0.98	0.99	0.99	1	0.99
**cAMPv**	*PC* of cAMP after adding glucose	mM	0.01	1e-8	983	0.98	0.99	0.99	0.99	1	1	0.99
**Cdc25**	*SSC* of Cdc25 before adding glucose	mM	0.01	1e-8	838	0.98	0.99	0.99	0.99	0.99	1	0.99
**Cdc25t**	Time to *PC* of Cdc25	Seconds	0.01	1	986	0.99	0.99	0.99	0.99	1	1	1
**Cdc25v**	*PC* of Cdc25 after adding glucose	mM	0.01	1e-8	951	0.98	0.99	0.99	0.99	0.99	1	0.99
**Gpa2at**	Time to *PC* of G- protein Gpa2a	Seconds	0.01	1	782	0.98	0.98	0.98	0.99	0.99	1	0.99
**Gpa2av**	*PC of* Gpa2a after adding glucose	mM	0.01	1e-8	890	0.98	0.99	0.99	0.99	1	1	0.99
**Krht**	Time to *PC* of Krh	Seconds	0.01	1	785	0.98	0.98	0.98	0.99	0.99	1	0.99
**Krhv**	*PC* of Krh after adding glucose	mM	0.01	1e-8	803	0.98	0.99	0.99	0.99	0.99	1	0.99
**Pde1t**	Time to *PC* of Pde1	Seconds	0.01	1	961	0.97	0.98	0.99	0.99	0.99	1	0.99
**Pde1v**	*PC* of Pde1 after adding glucose	mM	0.01	1e-8	724	0.98	0.98	0.99	0.99	0.99	1	0.99
**PKAi**	*SSC* of PKA before adding glucose	mM	0.01	1e-8	622	0.98	0.99	0.99	0.99	0.99	1	0.99
**PKAit**	Time to *PC* of Protein kinase A	Seconds	0.01	1	964	0.97	0.98	0.99	0.99	0.99	1	0.99
**PKAiv**	*PC* of PKA after adding glucose	mM	0.01	1e-8	998	0.98	0.99	0.99	0.99	0.99	1	0.99
**Ras2a**	*SSC* of Ras2a before adding glucose	mM	0.01	1e-8	846	0.98	0.99	0.99	0.99	0.99	1	0.99
**Ras2at**	Time to *PC* of Ras2a	Seconds	0.01	1	937	0.98	0.99	0.99	0.99	0.99	1	0.99
**Ras2av**	*PC* of Ras2a after adding glucose	mM	0.01	1e-8	865	0.98	0.99	0.99	0.99	1	1	0.99
