# Supplementary material for: Effect of Regulatory Architecture on Broad versus Narrow Sense Heritability
Source: PLoS Comput Biol. 2013 May 9;9(5):e1003053. doi: 10.1371/journal.pcbi.1003053 (PMC3649986; doi:10.1371/journal.pcbi.1003053)
Supplement: Table S7 — Summary of phenotype descriptions, variability thresholds and distribution of VA / VG ratios for the glycolysis model. The first three columns list the phenotype abbreviations used in this study, a text description of the phenotypes and their units. The thresholds used to filter out dataset with very low relative and/or absolute variability are listed in the next two columns, followed by the number of Monte Carlo simulations (out of 1000) passing the threshold. The last 7 columns contain quantiles and means of the VA/VG values for the datasets passing the variability threshold. (PDF) [file pcbi.1003053.s017.pdf]

Table S7. Summary of phenotypic values, variability thresholds and distribution of V_A/V_G ratios for the glycolysis model [20]**.** The first three columns list the phenotype abbreviations used in this study, a text description of the phenotypes and their units. The thresholds used to filter out datase with very low relative and/or absolute variability are listed in the next two columns, followed by the number of Monte Carlo simulations (out of 1000) passing the threshold. The last 7 columns contain quantiles and means of the V_A/V_G values for the datasets passing the variability threshold.

**Phenotype**	**Description**	**Units**	**Variability threshold**		**# of valid datasets**	**Quantiles and mean values of V_A/V_G**						
			**rel.**	**abs.**		**$Q_{0.05}$**	**$Q_{0.1}$**	**$Q_{0.2}$**	**$Q_{0.3}$**	**$Q_{0.5}$**	**$Q_{0.8}$**	**mean**
**ACE**	Steady state concentration *(SSC)* of acetaldehyde	mM	0.01	1e-4	728	0.90	0.94	0.98	0.99	1	1	0.98
**BPG**	*SSC* of bisphospoglycerate	mM	0.01	1e-4	478	0.67	0.72	0.81	0.90	0.96	0.98	0.90
**F16P**	*SSC* of fructose-1,6-bisphosphate	mM	0.01	1e-4	934	0.75	0.87	0.94	0.96	0.98	1	0.95
**F6P**	*SSC* of fructose 6-phosphate	mM	0.01	1e-4	908	0.83	0.89	0.95	0.96	0.98	1	0.96
**G6P**	*SSC* of glucose 6-phosphate	mM	0.01	1e-4	922	0.84	0.90	0.95	0.96	0.98	1	0.96
**GLCi**	*SSC* of internal glucose in cell	mM	0.01	1e-4	794	0.68	0.81	0.91	0.95	0.99	1	0.93
**NADH**	*SSC* of nicotinamide adenine dinucleotide	mM	0.01	1e-4	710	0.88	0.93	0.97	0.98	0.99	1	0.97
**P**	*SSC* of phosphates in adenine nucleotides	mM	0.01	1e-4	665	0.88	0.93	0.97	0.99	1	1	0.97
**P2G**	*SSC* of 2- phosphoglyerate	mM	0.01	1e-4	896	0.73	0.82	0.93	0.96	0.98	1	0.94
**P3G**	*SSC* of 3-phosphoglycerate	mM	0.01	1e-4	919	0.73	0.83	0.93	0.96	0.98	1	0.95
**PEP**	*SSC* of phosphoenolpyruvate	mM	0.01	1e-4	886	0.71	0.80	0.92	0.96	0.98	1	0.94
**PYR**	*SSC* of pyruvate	mM	0.01	1e-4	788	0.68	0.79	0.91	0.95	0.99	1	0.94
**TRIO**	*SSC* of trio-phosphate	mM	0.01	1e-4	851	0.77	0.88	0.95	0.97	0.99	1	0.96
