# Supplementary material for: Effect of Regulatory Architecture on Broad versus Narrow Sense Heritability
Source: PLoS Comput Biol. 2013 May 9;9(5):e1003053. doi: 10.1371/journal.pcbi.1003053 (PMC3649986; doi:10.1371/journal.pcbi.1003053)
Supplement: Table S8 — Summary of phenotype descriptions, variability thresholds and distribution of VA / VG ratios for the cell cycle model. The first three columns list the phenotype abbreviations used in this study, a text description of the phenotypes and their units. The thresholds used to filter out dataset with very low relative and/or absolute variability are listed in the next two columns, followed by the number of Monte Carlo simulations (out of 1000) passing the threshold. The last 7 columns contain quantiles and means of the VA/VG values for the datasets passing the variability threshold. (PDF) [file pcbi.1003053.s018.pdf]

Table S8. Summary of phenotypic values, variability thresholds and distribution of V_A/V_G ratios for the cell cycle model [22]**.** The first three columns list the phenotype abbreviations used in this study, a text description of the phenotypes and their units. The thresholds used to filter out dataset with very low relative and/or absolute variability are listed in the next two columns, followed by the number of Monte Carlo simulations (out of 1000) passing the threshold. The last 7 columns contain quantiles and means of the V_A/V_G values for the datasets passing the variability threshold.

Phenotype	Description	Units	Variability threshold		# of valid datasets	Quantiles and mean values of V_A/V_G						
			rel.	abs.		$Q_{0.05}$	$Q_{0.1}$	$Q_{0.2}$	$Q_{0.3}$	$Q_{0.5}$	$Q_{0.8}$	mean
**APCP**	Peak concentration (*PC*) of phosphorylated anaphase-promoting complex	au	0.01	1e-4	948	0.86	0.93	0.96	0.98	0.99	1	0.96
**APCP(ttp)**	Time to *PC* of APCP	min	0.01	0.5	726	0.92	0.95	0.97	0.98	0.99	1	0.98
**Bud**	Time of bud emergence	min	0.01	0.5	913	0.77	0.91	0.95	0.97	0.99	1	0.95
**Cdc6**	*PC* of Cdc6	au	0.01	1e-4	948	0.82	0.89	0.95	0.97	0.99	1	0.96
**Cdc6(ttp)**	Time to *PC* of Cdc6	min	0.01	0.5	836	0.83	0.86	0.92	0.94	0.97	0.99	0.95
**Clb2**	*PC* of B-type cyclin Clb2	au	0.01	1e-4	903	0.84	0.92	0.96	0.98	0.99	1	0.97
**Clb2(ttp)**	Time to *PC* of Clb2	min	0.01	0.5	749	0.94	0.96	0.97	0.98	0.99	1	0.98
**Clb5**	*PC* of B-type cyclin Clb5	au	0.01	1e-4	935	0.90	0.95	0.98	0.98	0.99	1	0.98
**Clb5(ttp)**	Time to *PC* of Clb5	min	0.01	0.5	779	0.90	0.93	0.96	0.97	0.99	1	0.97
**Cln2**	*PC* of cyclin Cln2	au	0.01	1e-4	914	0.78	0.90	0.95	0.97	0.99	1	0.96
**Cln2(ttp)**	Time to *PC* of Cln2	min	0.01	0.5	790	0.90	0.94	0.96	0.98	0.99	1	0.98
**Rep**	Start time of DNA replication	min	0.01	0.5	932	0.91	0.93	0.96	0.98	0.99	1	0.97
**Sic1**	*PC* of cyclin-dep. kinase inhibitor Sic1	au	0.01	1e-4	950	0.77	0.89	0.94	0.97	0.99	1	0.95
**Sic1(ttp)**	Time to *PC* of Sic1	min	0.01	0.5	751	0.82	0.84	0.89	0.92	0.96	0.99	0.93
**Spn**	Time of completed chromosome alignment	min	0.01	0.5	816	0.94	0.96	0.97	0.98	0.99	1	0.98
