# Supplementary material for: Effect of Regulatory Architecture on Broad versus Narrow Sense Heritability
Source: PLoS Comput Biol. 2013 May 9;9(5):e1003053. doi: 10.1371/journal.pcbi.1003053 (PMC3649986; doi:10.1371/journal.pcbi.1003053)
Supplement: Table S9 — Summary of phenotype descriptions, variability thresholds and distribution of VA / VG ratios for the circadian model. The first three columns list the phenotype abbreviations used in this study, a text description of the phenotypes and their units. The thresholds used to filter out dataset with very low relative and/or absolute variability are listed in the next two columns, followed by the number of Monte Carlo simulations (out of 1000) passing the threshold. The last 7 columns contain quantiles and means of the VA/VG values for the datasets passing the variability threshold. Abbreviations: phosphorylated – phos., cytosolic – cyt., nuclear – nuc., bottom concentration – b.c., peak concentration – p.c. (PDF) [file pcbi.1003053.s019.pdf]

Table S9. Summary of phenotypic values, variability thresholds and distribution of V_A/V_G ratios for the circadian model [23]**.** The first three columns list the phenotype abbreviations used in this study, a text description of the phenotypes and their units. The thresholds used to filter out dataset with very low relative and/or absolute variability are listed in the next two columns, followed by the number of Monte Carlo simulations (out of 1000) passing the threshold. The last 7 columns contain quantiles and means of the V_A/V_G values for the datasets passing the variability threshold. Abbreviations: phosphorylated – phos., cytosolic – cyt., nuclear – nuc., bottom concentration – b.c., peak concentration – p.c.

**Phenotype**	**Description (see legend for abbreviations)**	**Units**	**Variability threshold**		**# of valid datasets**	**Quantiles and mean values of V_A/V_G**						
			**rel.**	**abs.**		**$Q_{0.05}$**	**$Q_{0.1}$**	**$Q_{0.2}$**	**$Q_{0.3}$**	**$Q_{0.5}$**	**$Q_{0.8}$**	**mean**
**BC**	B.c. of cyt. BMAL1 protein	nM	0.01	1e-8	979	0.43	0.65	0.91	0.97	0.99	1	0.91
**BC(ttp)**	Time to p.c. of cyt. BMAL1 protein	hours	0.01	1e-8	997	0.40	0.70	0.85	0.91	0.96	0.98	0.88
**BCP**	B.c. of cyt. phos. BMAL1 protein	nM	0.01	1e-8	953	0.82	0.88	0.93	0.96	0.99	1	0.95
**BCP(ttp)**	Time to p.c. of cyt. phos. BMAL1 protein	hours	0.01	1e-8	999	0.62	0.80	0.87	0.92	0.97	0.99	0.91
**BN**	B.c. of nuc. BMAL1 protein	nM	0.01	1e-8	999	0.37	0.67	0.84	0.93	0.98	0.99	0.89
**BN(ttp)**	Time to p.c. of nuc. BMAL1 protein	hours	0.01	1e-8	1000	0.48	0.72	0.83	0.89	0.95	0.98	0.88
**BNP**	B.c. of nuc. Phos. BMAL1 protein	nM	0.01	1e-8	979	0.82	0.91	0.97	0.99	1	1	0.97
**BNP(ttp)**	Time to p.c. of nuc. Phos. BMAL1 protein	hours	0.01	1e-8	999	0.66	0.82	0.89	0.94	0.98	0.99	0.93
**CC**	B.c. of cyt. CRY protein	nM	0.01	1e-8	1000	0.59	0.63	0.69	0.74	0.82	0.95	0.81
**CC(ttp)**	Time to p.c. of cyt. CRY protein	hours	0.01	1e-8	1000	0.10	0.16	0.27	0.39	0.67	0.94	0.61
**CCP**	B.c. of cyt. phos. CRY protein	nM	0.01	1e-8	969	0.61	0.68	0.74	0.79	0.86	0.95	0.84
**CCP(ttp)**	Time to p.c. of cyt. phos. CRY protein	hours	0.01	1e-8	1000	0.10	0.15	0.31	0.46	0.70	0.91	0.62
**IN**	B.c. of inactive complex between CLOCK-BMAL1 and PER-CRY	nM	0.01	1e-8	1000	0.51	0.62	0.73	0.80	0.87	0.95	0.83
**IN(ttp)**	Time to p.c. of inactive complex between CLOCK-BMAL1 and PER-CRY	hours	0.01	1e-8	1000	0.22	0.36	0.53	0.66	0.82	0.95	0.73
**MB**	B.c. of mRNA of the *Bmal1* gene	nM	0.01	1e-8	901	0.32	0.50	0.79	0.93	0.99	1	0.88
**MB(ttp)**	Time to p.c. of mRNA of the *Bmal1* gene	hours	0.01	1e-8	894	0.19	0.53	0.78	0.82	0.88	0.95	0.81
**MC**	B.c. of mRNA of the *Cry* gene	nM	0.01	1e-8	999	0.91	0.92	0.94	0.96	0.98	0.99	0.96

**MC(ttp)**	Time to p.c. of mRNA of the *Cry* gene	hours	0.01	1e-8	1000	0.52	0.66	0.82	0.88	0.96	0.99	0.88
**MP**	B.c. of the mRNA of the *Per* gene	nM	0.01	1e-8	997	0.90	0.92	0.94	0.95	0.98	0.99	0.96
**MP(ttp)**	Time to p.c. of the mRNA of the *Per* gene	hours	0.01	1e-8	999	0.32	0.52	0.74	0.87	0.96	0.99	0.85
**PC**	B.c. of cyt. PER protein	nM	0.01	1e-8	1000	0.41	0.48	0.57	0.63	0.72	0.88	0.72
**PC(ttp)**	Time to p.c. of cyt. PER protein	hours	0.01	1e-8	1000	0.04	0.07	0.14	0.23	0.46	0.88	0.49
**PCC**	B.c. of cyt. PER-CRY complex	nM	0.01	1e-8	1000	0.8	0.83	0.87	0.90	0.93	0.97	0.92
**PCC(ttp)**	Time to p.c. of cyt. PER-CRY complex	hours	0.01	1e-8	1000	0.49	0.62	0.77	0.84	0.92	0.98	0.85
**PCCP**	B.c. of cyt. Phos. PER-CRY complex	nM	0.01	1e-8	1000	0.92	0.94	0.95	0.96	0.98	0.99	0.97
**PCCP(ttp)**	Time to p.c. of cyt. phos. PER-CRY complex	hours	0.01	1e-8	1000	0.46	0.61	0.75	0.83	0.92	0.98	0.85
**PCN**	B.c. of nuc. PER-CRY complex	nM	0.01	1e-8	1000	0.52	0.60	0.69	0.75	0.85	0.95	0.81
**PCN(ttp)**	Time to p.c. of nuc. PER-CRY complex	hours	0.01	1e-8	999	0.62	0.72	0.84	0.89	0.95	0.98	0.90
**PCNP**	B.c. of nuc. Phos. PER-CRY complex	nM	0.01	1e-8	1000	0.80	0.83	0.87	0.90	0.94	0.98	0.92
**PCNP(ttp)**	Time to p.c. of nuc. phos. PER-CRY complex	hours	0.01	1e-8	999	0.59	0.70	0.83	0.89	0.95	0.99	0.89
**PCP**	B.c. of cyt. phos. PER protein	nM	0.01	1e-8	989	0.70	0.75	0.81	0.85	0.91	0.96	0.88
**PCP(ttp)**	Time to p.c. of cyt. phos. PER protein	hours	0.01	1e-8	1000	0.04	0.07	0.15	0.24	0.46	0.88	0.50
**Period**	Time for one complete cycle	hours	0.01	0.1	997	0.60	0.81	0.90	0.94	0.98	0.99	0.92
