# Supplementary material for: Effect of Regulatory Architecture on Broad versus Narrow Sense Heritability
Source: PLoS Comput Biol. 2013 May 9;9(5):e1003053. doi: 10.1371/journal.pcbi.1003053 (PMC3649986; doi:10.1371/journal.pcbi.1003053)
Supplement: Table S10 — Summary of phenotype descriptions, variability thresholds and distribution of VA / VG ratios for the action potential model. The first three columns list the phenotype abbreviations used in this study, a text description of the phenotypes and their units. The thresholds used to filter out dataset with very low relative and/or absolute variability are listed in the next two columns, followed by the number of Monte Carlo simulations (out of 1000) passing the threshold. The last 7 columns contain quantiles and means of the VA/VG values for the datasets passing the variability threshold. (PDF) [file pcbi.1003053.s020.pdf]

Table S10. Summary of phenotypic values, variability thresholds and distribution of V_A/V_G ratios for the action potential model [24]**.** The first three columns list the phenotype abbreviations used in this study, a text description of the phenotypes and their units. The thresholds used to filter out dataset with very low relative and/or absolute variability are listed in the next two columns, followed by the number of Monte Carlo simulations (out of 1000) passing the threshold. The last 7 columns contain quantiles and means of the V_A/V_G values for the datasets passing the variability threshold.

Phenotype		Description	Units	Variability threshold		# of valid datasets	Quantiles and mean values of V_A/V_G						
				rel.	abs.		$Q_{0.05}$	$Q_{0.1}$	$Q_{0.2}$	$Q_{0.3}$	$Q_{0.5}$	$Q_{0.8}$	mean
Action Potential	25%	Time to 25% of the initial value	ms	0.01	1e-5	909	0.31	0.44	0.67	0.72	0.88	0.98	0.88
	50%	Time to 50% of the initial value	ms	0.01	1e-5	980	0.29	0.39	0.63	0.72	0.90	1	0.9
	75%	Time to 75% of the initial value	ms	0.01	1e-5	968	0.21	0.31	0.48	0.62	0.88	0.99	0.88
	90%	Time to 90% of the initial value	ms	0.01	1e-5	976	0.21	0.31	0.64	0.92	0.99	1	0.99
	Amplitude	Amplitude of action potential value	mV	0.01	1e-5	652	0.20	0.28	0.50	0.66	0.97	0.99	0.97
	Base	Initial action potential	mV	0.01	1e-5	1000	0.21	0.31	0.56	0.67	0.83	0.98	0.83
	Peak	Maximum action potential	mV	0.01	1e-5	892	0.19	0.27	0.45	0.59	0.77	0.99	0.77
	Time to peak	Time to maximum action potential	ms	0.01	1e-5	801	0.20	0.30	0.48	0.59	0.83	0.98	0.84
Calsium transient	25%	Time to 25% of the initial concentration	ms	0.01	1e-5	961	0.18	0.28	0.53	0.69	0.92	0.99	0.92
	50%	Time to 50% of the initial concentration	ms	0.01	1e-5	960	0.22	0.35	0.58	0.67	0.89	0.99	0.88
	75%	Time to 75% of the initial concentration	ms	0.01	1e-5	955	0.35	0.45	0.60	0.67	0.86	0.99	0.86
	90%	Time to 90% of the initial concentration	ms	0.01	1e-5	928	0.29	0.38	0.52	0.63	0.76	0.98	0.76
	Amplitude	Amplitude of the calcium concentration	uM	0.01	1e-5	990	0.18	0.29	0.46	0.58	0.77	0.98	0.77
	Base	Initial calcium concentration	uM	0.01	1e-5	965	0.27	0.36	0.51	0.63	0.78	0.99	0.78
	Peak	Maximum calcium concentration	uM	0.01	1e-5	983	0.28	0.36	0.52	0.61	0.73	0.98	0.73
	Time to peak	Time to maximum concentration	ms	0.01	1e-5	974	0.18	0.24	0.43	0.60	0.84	0.97	0.84
