# Supplementary material for: Effect of Regulatory Architecture on Broad versus Narrow Sense Heritability
Source: PLoS Comput Biol. 2013 May 9;9(5):e1003053. doi: 10.1371/journal.pcbi.1003053 (PMC3649986; doi:10.1371/journal.pcbi.1003053)
Supplement: Text S1 — More detailed descriptions of the five cGP models. (PDF) [file pcbi.1003053.s021.pdf]

Yunpeng Wang, Jon Olav Vik, Stig W. Omholt, Arne B. Gjuvsland (2013) Effect of Regulatory Architecture on Broad versus Narrow Sense Heritability. PLoS Comp Biol.

Text S1

Here we describe the five models in some more detail than in the main text.

cAMP Model

The complete cAMP signaling pathway [1] of *Saccharomyces cerevisiae*, involved in various essential cell activities such as nutrient sensing, stress response, growth, cell cycle progression, is modeled by a system of ordinary differential equations having 15 variables and 27 parameters. Glucose metabolism via the glycolysis pathway as well as the dynamics of the G-protein Gpa2a, the Kelch repeat homologue protein (Krh), the G-protein Ras2, and Protein Kinase A (PKA) is described by mass action kinetics. Modified Michaelis-Menten kinetics is used to describe the activity of adenylate cyclase and phosphodiesterase. A number of negative feedback mechanisms are included in the model. The model is capable of reproducing how cAMP levels change in response to addition of glucose both in wild-type and several mutant strains.

Glycolysis model

The branched kinetic model of the glycolysis pathway of non-growing anaerobic *Saccharomyces cerevisiae* [2] describes how external glucose molecules are transported into the cell, and conversion into pyruvate (ultimately to ethanol), glycogen, trehalose, glycerol, and succinate, by a series of enzyme-catalyzed reactions. The dynamics of 19 metabolic fluxes (12 reversible and 7 irreversible) and 17 intermediate concentrations, are represented by 14 ordinary differential equations. The model describes the kinetics of the 13 enzymes hexokinase (HK), phosphoglucose isomerase (PGI), phosphofructokinase (PFK), aldolase (ALD), triosephosphate isomerase (TPI), glycerol 3-phosphate dehydrogenase (G3PDH), glyceraldehyde-3-phosphate dehydrogenase (GraPDH), phosphoglycerate kinase (PGK), phosphoglycerate mutase (PGM), enolase (ENO), pyruvate kinase (PYK) pyruvate decarboxylase (PDC) and alcohol dehydrogenase (ADH).

Cell cycle model

A model based on the consensus mechanism of the cell cycle regulation in budding yeast [3]. The model describes this process by 36 ordinary differential and 25 algebraic equations. The four phases in cell cycle, S (the primary activity is DNA synthesis), G2 (preparing for mitosis), M (mitosis) and G1 (growth phase) are modeled as two states, the G1 state and the S-G2-M state. The unidirectional transitions between the two self-maintaining states are achieved by a bistable mechanism involving positive and negative feedback loops. The model is capable of mimicking the behaviour of >100 mutant strains.

Circadian model

The model describes a consensus network underlying the mammalian circadian clock [4,5], a regulatory network involving the *Per*, *Cry*, *Bmal1* and *Clock* genes. The model consists of 16 coupled ordinary differential equations, with intertwined positive and negative feedback loops. The state variable include mRNA, phosphorylated and unphorsphorylated proteins as well as protein complexes. A couple of sleep-wake related human disorders were studies with this model by examining the parameter spaces. The model gives rice to sustained oscillation with a period close to 24 hours under continuous darkness.

Action potential model

The mouse ventricular myocyte model [6] extends that of Bondarenko et al [7] with more realistic calcium handling, detailed re-parameterization to consistent experimental data for the C57BL/6 "black 6" mouse and conservation of charge. State variables include ion concentrations of sodium, potassium and calcium in the cytosol, calcium concentration in the sarcoplasmic reticulum, and the conformation state distribution of ion channels, whose transition rates between open, closed, and inactivated conformations may depend on transmembrane voltage. Formulated as a system of 35 coupled ordinary differential equations with more than hundred of parameters, this model provides a comprehensive representation of membrane-bound channels and transporter functions as well as fluxes between the cytosol and intracellular organelles.

1. Williamson T, Schwartz J-M, Kell DB, Stateva L (2009) Deterministic

mathematical models of the cAMP pathway in Saccharomyces cerevisiae. BMC Syst Biol 3: 70. doi:10.1186/1752-0509-3-70.

2. Teusink B, Passarge J, Reijenga CA, Esgalhado E, van der Weijden CC, et al. (2000) Can yeast glycolysis be understood in terms of in vitro kinetics of the constituent enzymes? Testing biochemistry. Eur J Biochem 267: 5313–5329.

3. Chen KC, Calzone L, Csikasz-Nagy A, Cross F, Novak B, et al. (2004) Integrative Analysis of Cell Cycle Control in Budding Yeast. Molecular Biology of the Cell 15: 3841–3862. doi:10.1091/mbc.E03-11-0794.

4. Leloup J-C, Goldbeter A (2003) Toward a detailed computational model for the mammalian circadian clock. Proc Natl Acad Sci USA 100: 7051–7056. doi:10.1073/pnas.1132112100.

5. Leloup J-C, Goldbeter A (2004) Modeling the mammalian circadian clock: sensitivity analysis and multiplicity of oscillatory mechanisms. J Theor Biol 230: 541–562. doi:10.1016/j.jtbi.2004.04.040.

6. Li L, Niederer SA, Idigo W, Zhang YH, Swietach P, et al. (2010) A mathematical model of the murine ventricular myocyte: a data-driven biophysically based approach applied to mice overexpressing the canine NCX isoform. Am J Physiol Heart Circ Physiol 299: H1045–H1063. doi:10.1152/ajpheart.00219.2010.

7. Bondarenko VE, Szigeti GP, Bett GCL, Kim S-J, Rasmusson RL (2004) Computer model of action potential of mouse ventricular myocytes. Am J Physiol Heart Circ Physiol 287: H1378–H1403. doi:10.1152/ajpheart.00185.2003.
